# Induction of Mitosis Delay and Apoptosis by CDDO-TFEA in Glioblastoma Multiforme

**DOI:** 10.3389/fphar.2021.756228

**Published:** 2021-11-08

**Authors:** Tai-Hsin Tsai, Ann-Shung Lieu, Tzuu-Yuan Huang, Aij-Lie Kwan, Chih-Lung Lin, Yi-Chiang Hsu

**Affiliations:** ^1^ Division of Neurosurgery, Department of Surgery, Kaohsiung Medical University Hospital, Kaohsiung, Taiwan; ^2^ Department of Surgery, School of Medicine, College of Medicine, Kaohsiung Medical University, Kaohsiung, Taiwan; ^3^ Graduate Institutes of Medicine, College of Medicine, Kaohsiung Medical University, Kaohsiung, Taiwan; ^4^ Department of Neurosurgery, Changhua Christian Hospital, Changhua, Taiwan; ^5^ School of Medicine, I‐Shou University, Kaohsiung, Taiwan

**Keywords:** CDDO-TFEA, GBM, cell cycle, RTA 404, apoptosis

## Abstract

**Background:** Glioblastoma multiforme (GBM) is the vicious malignant brain tumor in adults. Despite advances multi-disciplinary treatment, GBM constinues to have a poor overall survival. CDDO-trifluoroethyl-amide (CDDO-TEFA), a trifluoroethylamidederivative of CDDO, is an Nrf2/ARE pathway activator. CDDO-TEFEA is used to inhibit proliferation and induce apoptosis in glioma cells. However, it not clear what effect it may have on tumorigenesis in GBM.

**Methods:** This *in vitro* study evaluated the effects of CDDO-TFEA on GBM cells. To do this, we treated GBM8401 cell lines with CDDO-TFEA and assessed apoptosis, cell cycle. DNA content and induction of apoptosis were analyzed by flow cytometry and protein expression by Western blot analysis.

**Results:** CDDO-TFEA significantly inhibited the cell viability and induced cell apoptosis on GBM 8401 cell line. The annexin-FITC/PI assay revealed significant changes in the percentage of apoptotic cells. Treatment with CDDO-TFEA led to a significant reduction in the GBM8401 cells’ mitochondrial membrane potential. A significant rise in the percentage of caspase-3 activity was detected in the treated cells. In addition, treatment with CDDO-TFEA led to an accumulation of G_2_/M-phase cells. In addition, these results suggest that regarding increased protein synthesis during mitosis in the MPM-2 staining, indicative of a delay in the G2 checkpoint. An analysis of Cyclin B1, CDK1, Cyclin B1/CDK1 complex and CHK1 and CHK2 expression suggested that cell cycle progression seems also to be regulated by CDDO-TFEA. Therefore, CDDO-TFEA may not only induce cell cycle G2/M arrest, it may also exert apoptosis in established GBM cells.

**Conclusion:** CDDO-TFEA can inhibit proliferation, cell cycle progression and induce apoptosis in GBM cells *in vitro*, possibly though its inhibition of Cyclin B1, CDK1 expression, and Cyclin B1/CDK1 association and the promotion of CHK1 and CHK2 expression.

## 1 Introduction

Glioma, the most common type of brain tumor, is classified by the World Health Organization (WHO) into four grades based on histology features (Bush et al., 2017; Cahill and Turcan, 2018). Glioblastoma, also called glioblastoma multiforme (GBM), is an angiogenic, aggressive grade IV glioma of the central nervous system that can cause necrosis. Despite advances in multidisciplinary treatment, overall survival rates remain low. Patients with glioblastoma have a median survival rate of approximately 12 months (Zachariah et al., 2018). Therefore, identifying new and effective anticancer drugs and understanding their mechanism of action in treating malignant tumors is essential.

A component of Chinese herbal medicine prescribed for hepatitis, oleanolic acid has been chemically modified to create an oleanane triterpenoid, 2-cyano-3-,12-dioxoolean-1,9-dien-28-oic acid (CDDO) (Gao et al., 2019). The therapeutic effect of CDDO stems from its ability to upregulate nuclear erythroid 2-related factor 2 (Nrf2) by changing the conformation of the Nrf2-repressing Kelch-like erythroid cell-derived protein with cap’n’collar homology associated protein 1 (Keap1) (Wang et al., 2015). Several animal and human studies have determined that the antioxidant response element activates Nrf2-controlled antioxidant genes upstream (Wang et al., 2015). A considerable amount of research has been devoted to manipulating these compounds to generate new derivatives with more desirable traits, including by increasing their anti-inflammatory activity and creating additional functional groups for symptom treatment. CDDO derivatives have been used to treat lung injury (Wang et al., 2015), inflammation (Shanmugam et al., 2014), and chronic kidney disease (Wang et al., 2014a). Mounting evidence indicates that CDDO and its derivatives have anticancer effects. Because of their strong antiproliferative, antiangiogenic, and antimetastatic activities, CDDO and its derivatives bardoxolone methyl and CDDO-imidazolide have been studied extensively for the potential ability to induce apoptosis and differentiation in cancer cells (Borella et al., 2019).

CDDO–trifluoroethyl amide (CDDO–TFEA), a trifluoroethylamide derivative of CDDO, has antioxidation, anti-inflammatory, and antiproliferative effects, in addition to an enhanced ability to cross the blood–brain barrier (Yang et al., 2020). Currently, CDDO-TFEA has been used to treat neurodegenerative diseases, neuroinflammatory diseases, and neurooncology cancers. It also enhances Nrf2 expression and signaling in various models of neurodegeneration (Gupta et al., 2012), including those that simulate multiple sclerosis (Pareek et al., 2011), amyotrophic lateral sclerosis (Neymotin et al., 2011), and Huntington’s disease (Stack et al., 2010). CDDO-TFEA has exerted neuroprotective effects in some animal models of degenerative conditions such as ischemic stroke (Yang et al., 2020) and autoimmune encephalomyelitis (Pareek et al., 2011). Moreover, in one study, it induced apoptosis in neuroblastoma cells (Alabran et al., 2008). In addition, in GMC cells, CDDO-TFEA can inhibit proliferation, cell locomotion, and cell cycle progression and induce apoptosis (Tsai et al., 2021).

Although CDDO-TFEA has been found to inhibit some cancers (Wang et al., 2014b), its effect on GBM and the route through which it may achieve this effect remain unclear. The present study focuses on CDDO-TFEA and its chemopreventive activities against glioma cells, specifically GBM8401 cells, malignant glioma cells found in the human brain. We examined CDDO-TFEA’s effects on cell growth and cell cycle regulation and evaluated the expression levels of downstream molecules. Results demonstrate that CDDO-TFEA reduced glioma cell proliferation and induced apoptosis in GBM8401 cells. Furthermore, the increase in the populations of GBM8401 cells in the G2 and M phases can be attributed to the repression of cyclin B and CDK1 after their exposure to CDDO-TFEA. The novelty of this study is that CDDO-TFEA, a novel and potential anticancer drug, induces cell apoptosis and induces cycle arrest in GBM.

## 2 Results

### 2.1 CDDO-TFEA Reduces the Viability of GBM8401 Cells

**FIGURE 1:**
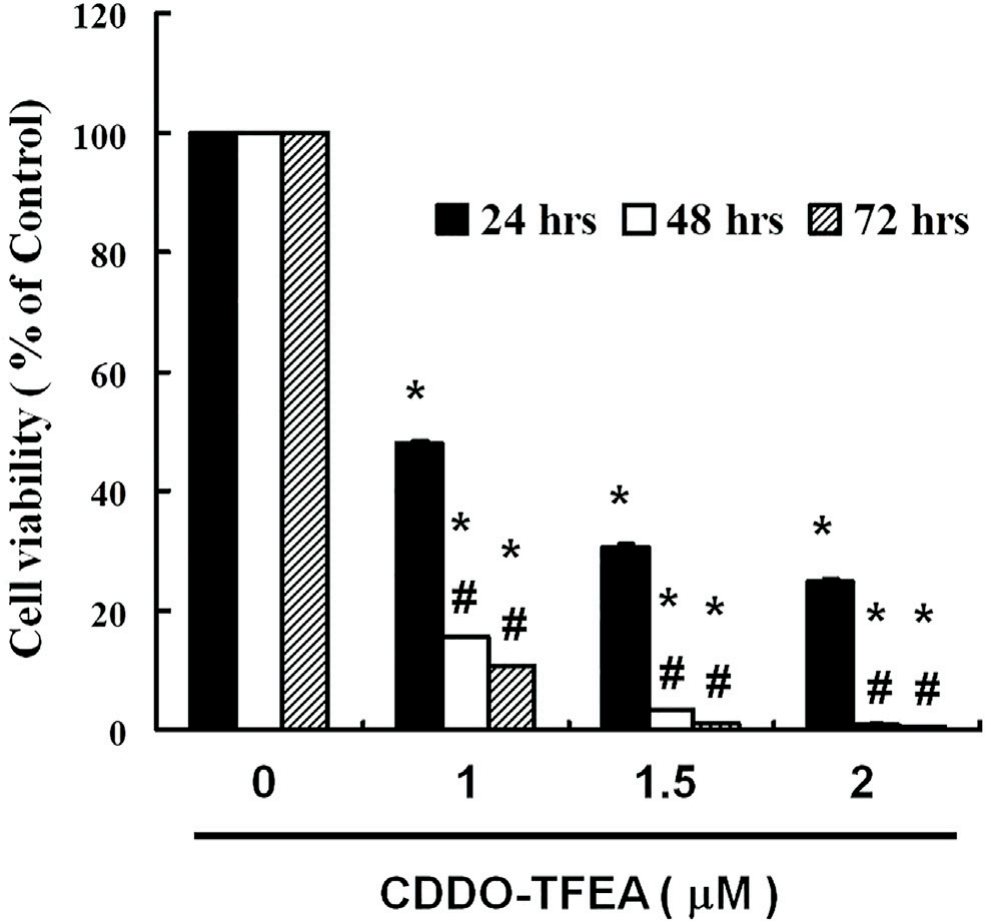
Viability of GBM8401 cells after 24–72 h of CDDO-TFEA treatment. CDDO-TFEA mediated the survival of GBM8401 cells by inhibiting proliferation. Cells were treated with increasing doses of CDDO-TFEA for 24–72 h *in vitro*. The survival rate of the treated cancer cells was measured using a PB assay. Results are expressed as percentages of the control, with the control being 100%. All data are reported as means ± standard errors of the mean on the basis of the results of at least three separate experiments. Statistical analysis was performed using the *t* test, with differences between the treatment and control groups (# vs. 24-h treatment) considered significant at *p* < 0.05, as denoted by * and # standard errors of the mean.

To determine whether CDDO-TFEA mediates the cell viability of GBM8401 cell *s,* we treated each glioma cell line with increasing doses of CDDO-TFEA (0, 1, 1.5, and 2 μM) for 24 h and then measured cell viability by using peripheral blood (PB). We use normal lung fibroblasts MRC-5 cells as a control group for viability. There was no change in normal lung fibroblasts MRC-5 cells. As shown in Figure 1, the cell viability of the GBM8401 cells decreased significantly inversely with the number of CDDO-TFEA doses (24 h: y = −24.339x + 111.61, R^2^ = 0.841; 48 h: y = −30.959x + 107.27, R^2^ = 0.7175; 72 h: y = −30.813x + 105.13, R^2^ = 0.6821) administered over 24–72 h.

### 2.2 CDDO-TFEA Induced Apoptosis in GBM8401 Cells

**FIGURE 2:**
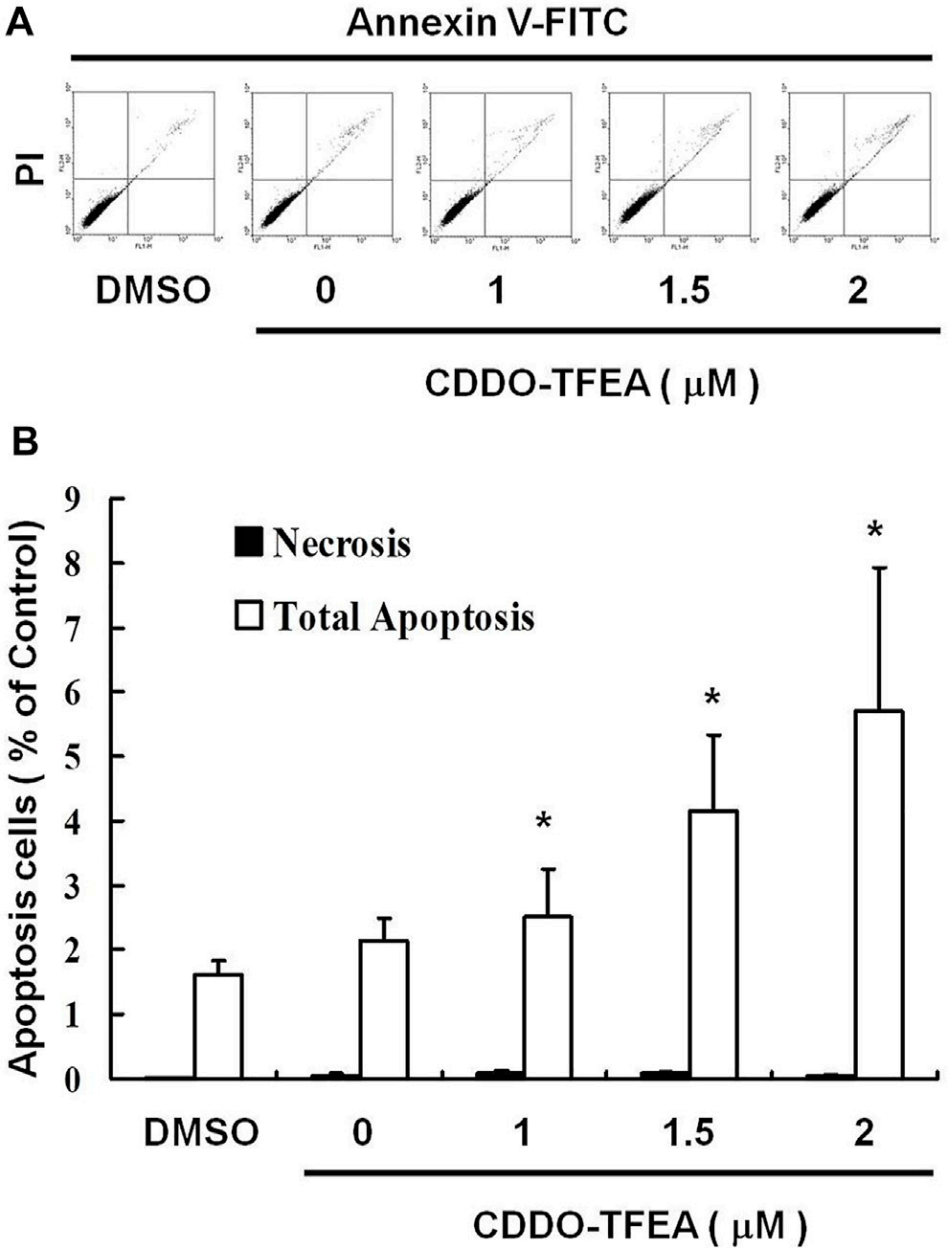
CDDO-TFEA induced apoptosis in GBM8401 cells. **(A)** Annexin V-FITC and **(B)** Propidium Iodide (PI) staining. Results are expressed as a percentage of the control, with the control being 100%. All data are reported as means ± standard errors of the mean on the basis of the results of at least three separate experiments. Statistical analysis was performed using the *t* test, with differences between the treatment and control groups considered significant at *p* < 0.05, as denoted by *.

To elucidate the role of CDDO-TFEA in the apoptosis of GBM8401 cells, we treated the cells with CDDO-TFEA for 4 h. This was followed by annexin V-FITC detection and Propidium Iodide (PI) staining, the caspase-3 assay, and the JC-1 assay. Cell populations and apoptotic ratios were analyzed through flow cytometry. The annexin-FITC/PI assay revealed significant changes in the percentage of apoptotic cells under and not under CDDO-TFEA treatment (i.e., the control cells; Figure 2A). The percentage of apoptotic cells increased significantly in the treated cells relative to in the untreated cells (Figure 2B; y = 1.0137x + 0.187; R2 = 0.9202).

**FIGURE 3:**
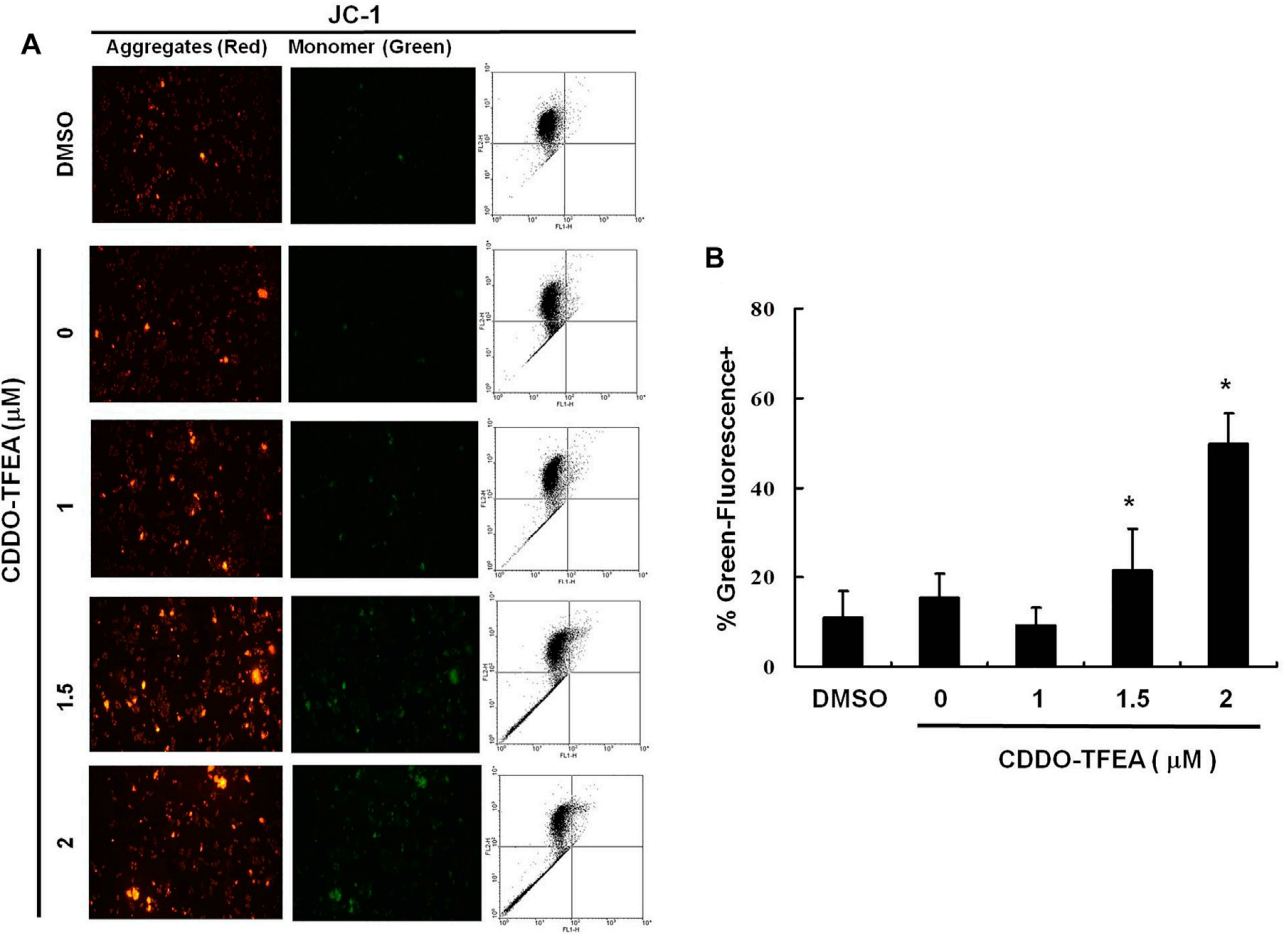
The disruption of mitochondrial membrane potential due to CDDO-TFEA treatment. **(A)** JC-1 was also *detected by using* fluorescence microscopy and flow cytometry. **(B)** Results are expressed as percentage of the control, control being 100%. All data are reported as the mean (±SEM) based on results of at least three separate experiments. Statistical analysis was performed using the t test, with differences between the treatment and control groups (0 M CDDO-TFEA) considered significant at *p* < 0.05, delineated by *.

The loss of mitochondrial membrane potential (ΔΨm), an early cellular metabolism event coinciding with caspase activation, is a hallmark of apoptosis. In nonapoptotic cells, JC-1 is present as a green monomer in the cytosol, but it accumulates as red aggregates in the mitochondria. In apoptotic and necrotic cells, although JC-1 is present in a monomeric form, it stains the cytosol green. To explore the possible effect of CDDO-TFEA on ΔΨm in the GBM cells, we used JC-1 as a dye to assess ΔΨm loss in the treated cancer cells. As shown in the left panel of Figure 3A, the cells’ ΔΨm was reduced after treatment with CDDO-TFEA. The right panel of Figure 3A presents typical FL-1/FL-2 dot plots for apoptotic and nonapoptotic GBM8401 cells stained with JC-1. The untreated cancer cells did not experience apoptosis, resulting in red fluorescing JC-1 aggregates. The green fluorescing monomers in the lower part of the figure indicate apoptotic cells. In sum, treatment with CDDO-TFEA led to a significant reduction in the GBM8401 cells’ ΔΨm (Figure 3B, y = 11.482x − 4.7167; R2 = 0.6904). The results displayed in Figures 2, 3 suggest that CDDO-TFEA mediates the survival of the GBM8401 cell line. Thus, we postulated that the proliferation of these cells was inhibited by apoptosis pathways.

**FIGURE 4:**
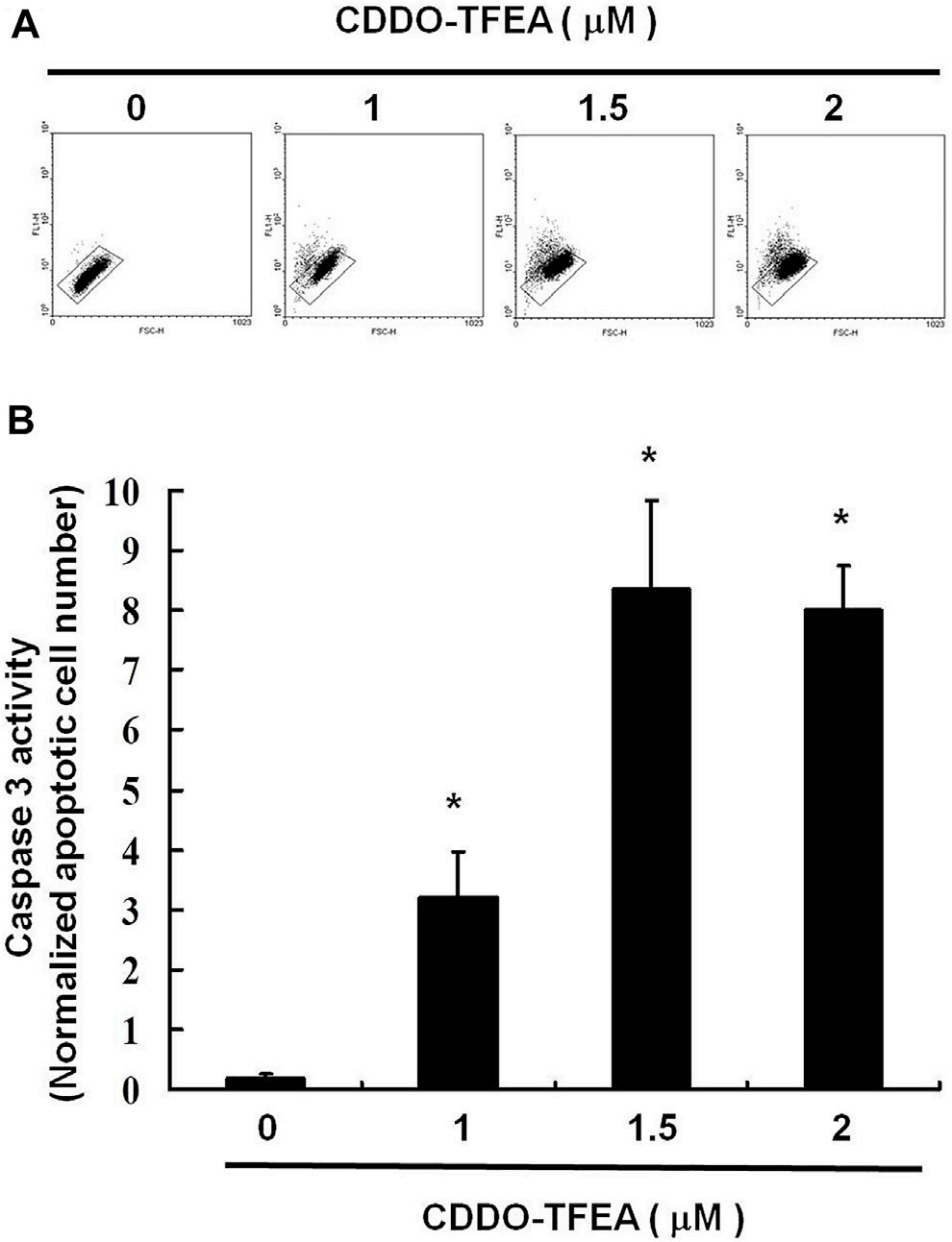
**(A)** Numbers of active caspase-3 induced by CDDO-TFEA treatment on the GBM8401 cell line. **(B)** Results are expressed as a percentage of the control, with the control being 100%. All data are reported as the means and standard errors of the mean on the basis of the results of at least three separate experiments. Statistical analysis was performed using the *t* test, with differences between the treatment and control groups considered significant at *p* < 0.05, as denoted by *.

A significant rise in the percentage of caspase-3 activity was detected in the treated cells (Figures 4A,B, y = 2.863x − 2.2267; R2 = 0.8766). This indicates that incubation with CDDO-TFEA induced cell apoptosis.

### 2.3 CDDO-TFEA Induced Cell Cycle Arrest in the G2/M Phase in GBM8401 Cells

**FIGURE 5:**
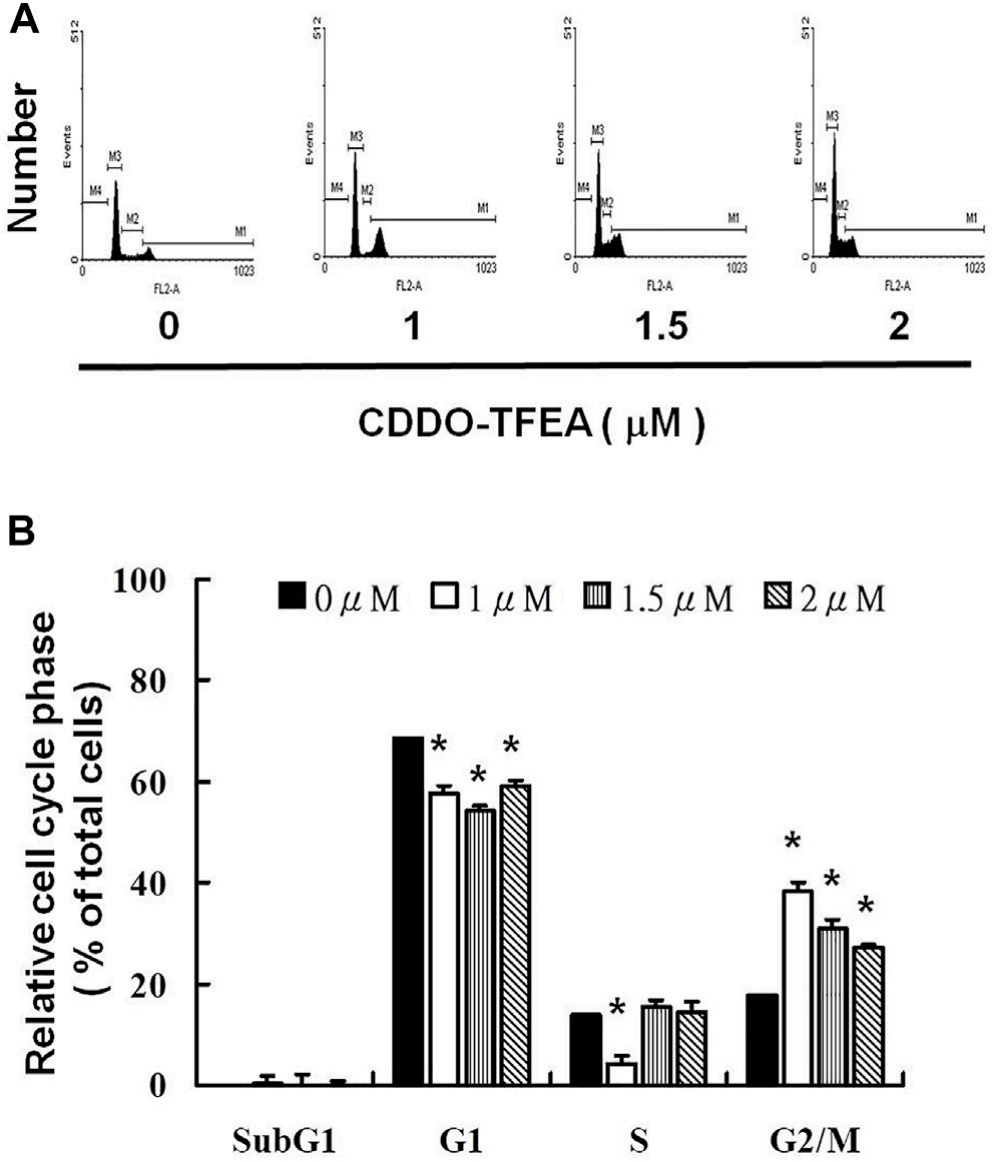
**(A)** Cell cycle arrest of GBM8401 cells in the G2/M phase caused by CDDO-TFEA treatment. Cell cycle analysis was conducted after 24 h of culture with CDDO-TFEA. CDDO-TFEA induced an increase in the proportion of cells in the G2/M phase. Propidium Iodide (PI) staining was conducted for DNA content analysis. Quantification was then performed through flow cytometry panel **(B)**. In each group of bars, * indicates that the number of G2/M cells in the treatment group was significantly higher than that in the control group (*p* < 0.05).

To further investigate the effect of CDDO-TFEA on cell growth, we analyzed and quantified the cell cycle distribution among the treated cells through flow cytometry. Specifically, cells were treated with CDDO-TFEA for 24 h. This was followed by processing and analysis. As shown in Figure 5A, treatment with CDDO-TFEA led to an incremental increase in the population of cells in the G2/M phase, indicative of a delay in the G2/M checkpoint. Overall, exposure to CDDO-TFEA increased the populations of cells in the G2/M phase (Figure 5B).

### 2.4 Failure to Pass the G2 Checkpoint Leads to Cell Cycle Arrest in the G2/M Phase in GBM8401 Cells

**FIGURE 6:**
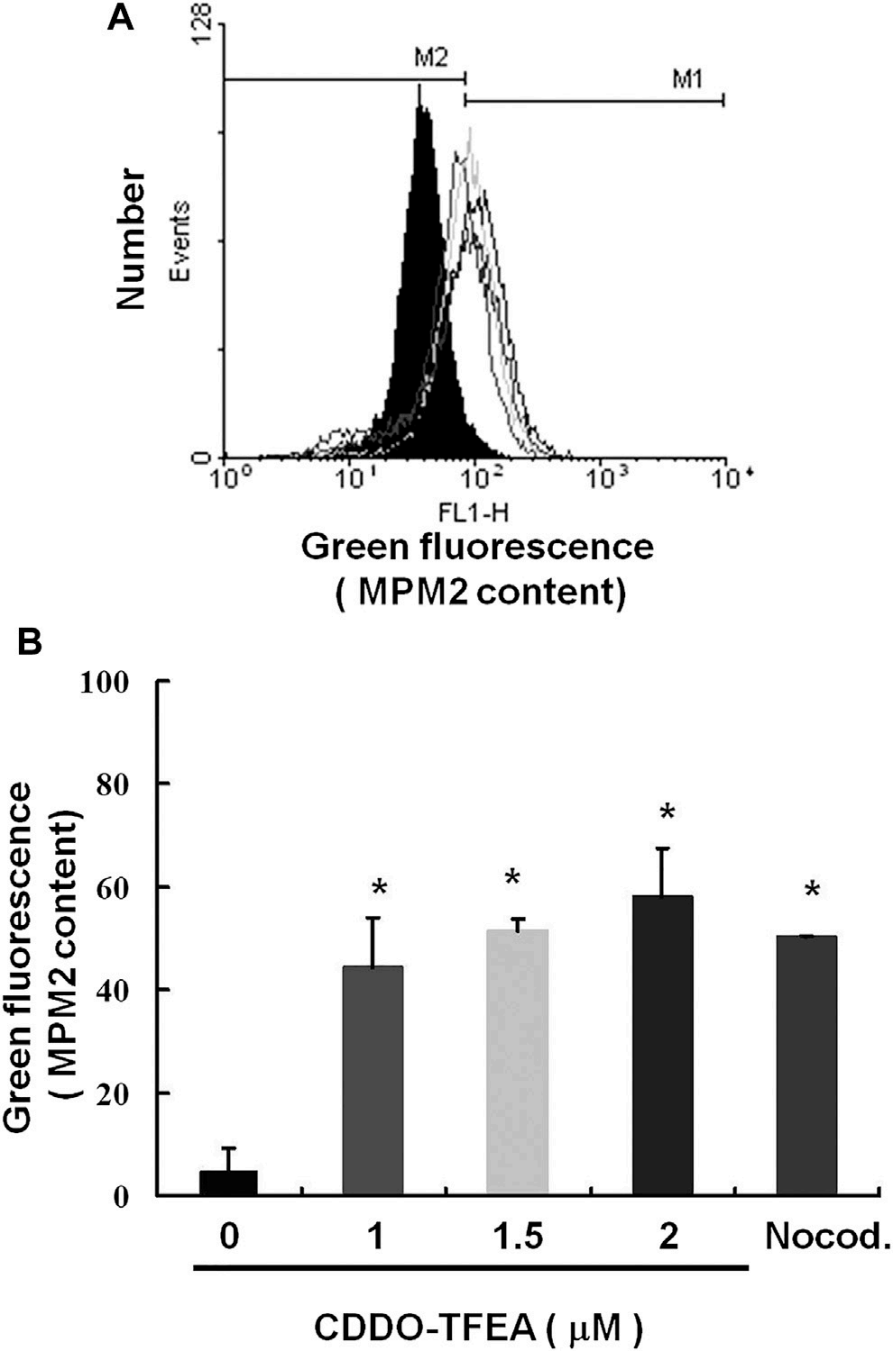
Cell failure to pass the G2 checkpoint leads to cell cycle arrest in the G2/M Phase in GBM8401 Cells. Flow cytometry analysis of MPM-2 expression in the treated cells was conducted. **(A)** Cells were either treated or not treated with 0, 1, 1.5, or 2 μM CDDO-TFEA. After 24 h of treatment, cells were fixed with 70% ethanol, stained with MPM-2 and Propidium Iodide (PI), and analyzed using FACScan software. **(B)** Results are expressed as a percentage of the control, with the control being 100%. All data are reported as means ± standard errors of the mean on the basis of the results of at least three separate experiments. Statistical analysis was performed using the *t* test, with differences between the treatment and control groups considered significant at *p* < 0.05, as denoted by *.

We explored the potential role of CDDO-TFEA treatment in mitosis in the GBM8401 cells. Protein synthesis during mitosis of the treated cells was analyzed through MPM-2 staining. Nocodazole is an anti-mitotic agent that reversibly interferes with the polymerization of microtubules. Nocodazole induce cell G2 phase arrest as positive control. Figure 6 displays the number of cells exposed to CDDO-TFEA at various concentrations (0, 1, 1.5, and 2 μM). Figures 6A,B demonstrate that exposure to CDDO-TFEA increased levels of protein synthesis during mitosis (y = 16.635x − 1.955; R2 = 0.8103). These results suggest that regarding increased protein synthesis during mitosis in the MPM-2 staining. It is implied that cell failure to pass the G2 checkpoint leads to cell cycle arrest in the G2/M Phase in GBM8401 Cells.

### 2.5 CDDO-TFEA Inhibited Cell Cycle Arrest Through the Cell Cycle Relates Protein

**FIGURE 7:**
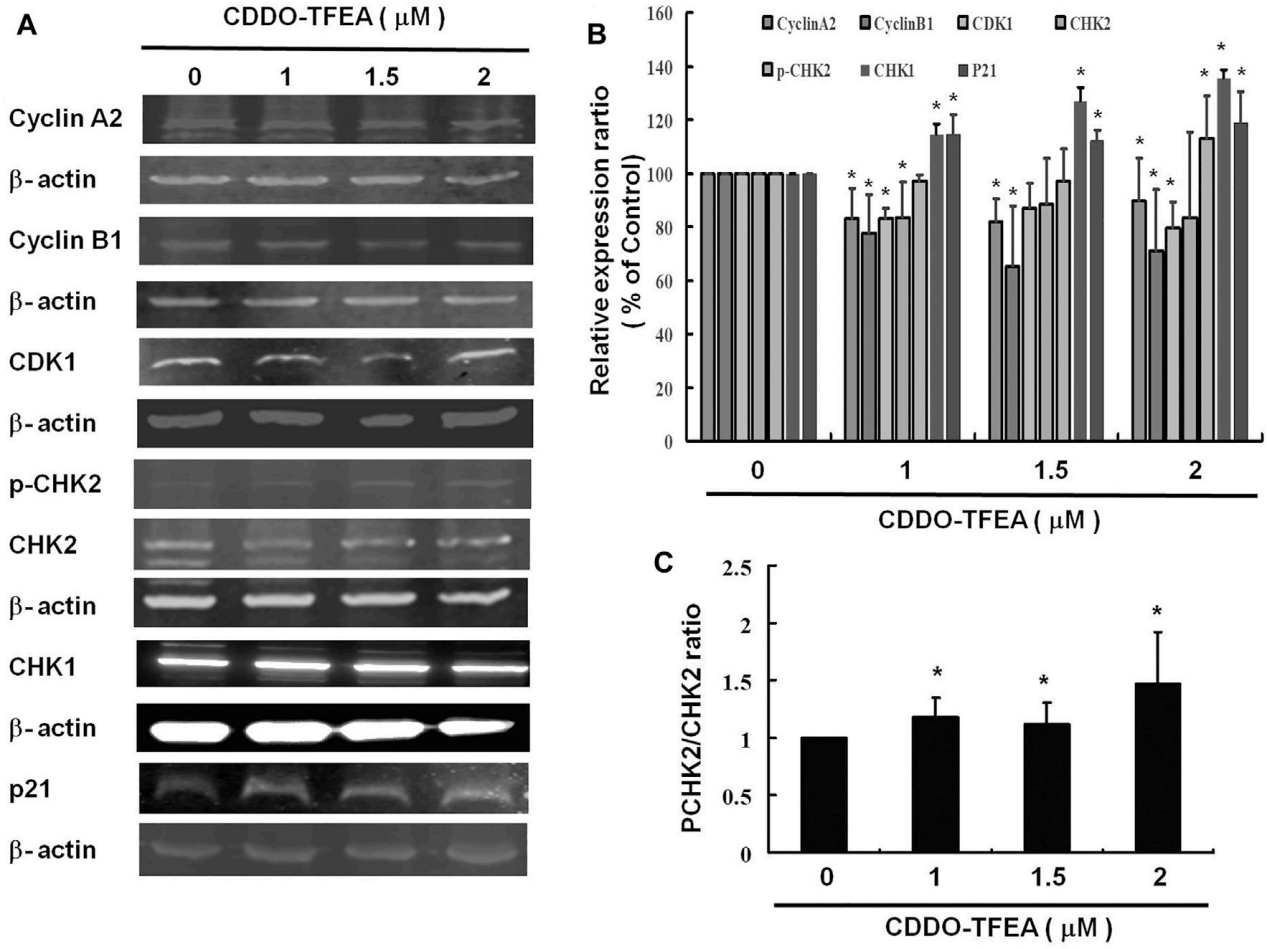
**(A)** CDDO-TFEA regulated cell cycle–related gene expression in cyclin A2, cyclin B1, and CDK1, as well as apoptosis- (p21-) related gene expression in the GBM8401 cells. Cells were treated with CDDO-TFEA for 24 h. Gene and protein expression was subsequently detected through Western blotting. **(B)** Results are expressed as a percentage of the control, with the control being 100%. All data are reported as means ± standard errors of the mean on the basis of the results of at least three separate experiments. Statistical analysis was performed using the *t* test, with differences between the treatment and control groups considered significant at *p* < 0.05, as denoted by *.

CDDO-TFEA induced apoptosis and inhibited cell cycle arrest in the GBM cells. Figures 7A,B displays the results of the Western blotting analysis of cell proteins extracted from the treated cells. In this experiment, we measured the relative intensities of cell cycle regulators such cyclin A2, cyclin B1, CDK1 expression. As Figure 7 shown, the relative intensities of Cyclin A2, cyclin B1 and CDK 1 were significantly downregulated after CDDO-TFEA treatment. In addition, we also measured the relative intensities of human checkpoint kinase such as CHK1, CHK2 and p-CHK2 expression. The relative intensity of CHK2 was a without significantly difference, but the relative intensity of CHK1 and the ratio of p-CHK2 and CHK2 (Figure 7C) were significantly upregulated. Furthermore, we measured the relative intensities of cyclin-dependent kinase inhibitor1, p21 expression. The intensity of p21 was significantly upregulated.

**FIGURE 8:**
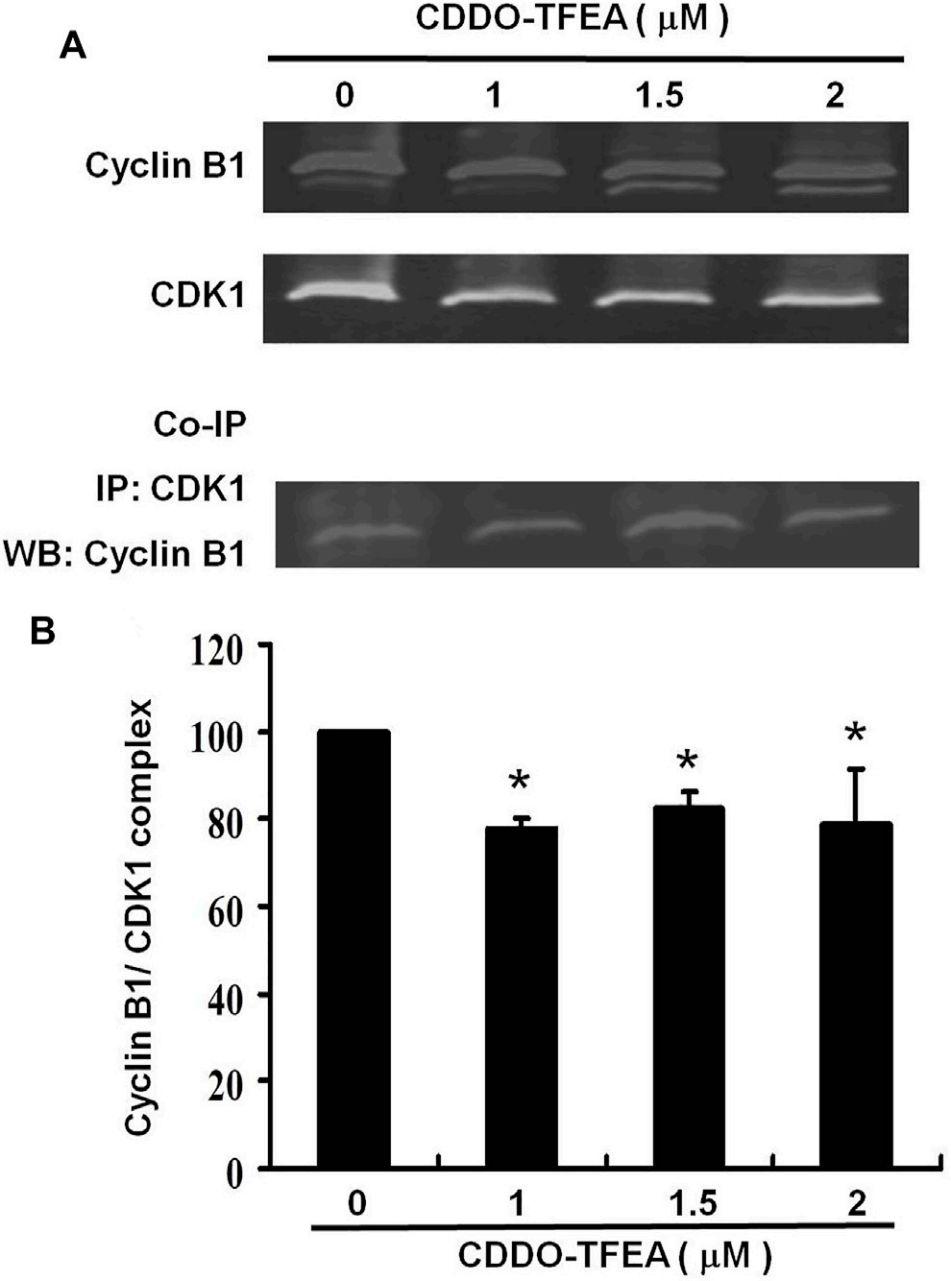
Co-Immunoprecipitation is an effective means of quantifying protein–protein interactions in cells. The primary and secondary targets were CDK1 and cyclin B1, respectively. Cells were treated with CDDO-TFEA for 24 h. Gene and protein expression was detected through Western blotting. **(B)** Results are expressed as a percentage of the control, with the control being 100%.

Co-Immunoprecipitation is an effective means of quantifying protein–protein interactions in cells (Figure 8A). They were found to cause significant reductions in cyclin B1/CDK1 complexes (Figure 8B) in treated cells (y = −5.8352x +99.362; R2 = 0.5293). These results suggest that regarding cell failure to pass the G2 Checkpoint, G2/M arrest may occur through cyclin B1, CDK1, and cyclin B1/CDK1complexes.

## 3 Discussion

CDDO-TFEA reduced the proliferation of and induced apoptosis in GBM8401 cells. Moreover, it led to an accumulation of cells in the G2/M phase. Cell cycle progression also appears to be regulated by CDDO-TFEA through the inhibition of cyclin B1, CDK1 and cyclin B1/CDK1 complexes and the promotion of CHK1 and p-CHK2 expression. Therefore, CDDO-TFEA may not only induce cell cycle arrest but also induce apoptosis in established GBM cells.

### 3.1 CDDO-TFEA Induced Apoptosis Through Intrinsic Apoptotic Pathway

Induction of apoptosis and arrest of the cell cycle are two of the best approaches for suppressing cancerous tumors (Saleem et al., 2018). Apoptosis is a process of programmed cell death that is involved in intrinsic and extrinsic pathways that activate the caspase family of cysteine proteases. Apoptotic markers such as BAX induce apoptosis through ΔΨm alteration that results in the translocation of cytochrome c from the mitochondrial inner membrane to the cytosol that triggers the signaling of caspase cascades (Liu et al., 1996; Kluck et al., 1997). Cytochrome c activates and causes cleaving in caspase-9, which in turn activates caspase-3 (Li et al., 1997). Caspase-9 activates downstream caspase-3 (Li et al., 1997), a well-known apoptotic mediator that drives DNA damage and subsequently apoptosis.

In the extrinsic pathway, stimulation of the TNF family of receptors results in the activation of caspase-8 (Boldin et al., 1996), which can directly activate caspase-3 (Stennicke et al., 1998). Subsequently, activation of the same target apoptotic molecules such as caspase-3 occurs (Jeong and Seol, 2008). Various anticancer drugs induce apoptosis by activating intrinsic pathways (Herr and Debatin, 2001). Available evidence indicates that CDDO induces apoptosis by activating the extrinsic caspase-8 pathway (Ito et al., 2000; Ito et al., 2001; Pedersen et al., 2002; Stadheim et al., 2002; Ikeda et al., 2003).

In the present study, CDDO-TFEA induced cytotoxicity in GBM8401 cells, and no change occurred in Hs-68 cells (normal skin fibroblasts) or MRC-5 cells (normal lung fibroblasts). CDDO-TFEA reduced the viability of GBM8401 cells by inducing apoptosis, as demonstrated by the dose-dependent increase in the number of annexin V–positive cells and the observation of nuclear chromatin condensation and DNA fragmentation therein following incubation with CDDO-TFEA (Figure 2). Moreover, a significant rise in the proportion of caspase-3 activity was detected in the treated cells (Figure 3). As revealed through fluorescent microscopy, CDDO-TFEA also reduced the aggregate-to-monomer ratio of JC-1, corresponding to the reduced number of cells, with no loss in ΔΨm (Figure 4). Therefore, according to the effects of CDDO-TFEA in the upregulation of caspase-3 and the alteration of ΔΨm, we can conclude that CDDO-TFEA induced apoptosis in the GBM8401 cells via an intrinsic pathway.

### 3.2 CDDO-TFEA Induced Cell Cycle Arrest in the G2/M Phase

Cell cycle deregulation, a feature unique to human cancer, involves the mutation of cell cycle–regulated genes, which allows a cell to circumvent checkpoint control systems (Hanahan and Weinberg, 2011). Treatment with CDDO-TFEA in various types of cell lines can induce cell cycle arrest in different phases. Alabran et al. observed an increase in cells in the sub-G1/G0 phase and a considerable decline in cells in the S-phase following treatment with CDDO-TFEA. The severe depletion of S-phase cells indicates that DNA synthesis is extremely sensitive to CDDO-TFEA treatment. However, because cells trapped in the G2/M phase during triterpenoid treatment did not progress into the G1/G0 phase, the researchers could not exclude the possibility of a G2/M block in addition to the S-phase depletion (Alabran et al., 2008). Malignant human glioma cell lines incubated with CDDO-TFEA resulted in cell cycle arrest in the G2/M phase (Tsai et al., 2021). As shown in Figure 5, treatment with CDDO-TFEA led to an incremental increase in the population of cells in the G2/M phase, In addition, these results suggest that regarding increased protein synthesis during mitosis in the MPM-2 staining, indicative of a delay in the G2/M checkpoint. Overall, exposure to CDDO-TFEA failure to pass the G2 checkpoint leads to cell cycle arrest in the G2/M Phase in GBM8401 Cells.

### 3.3 CDDO-TFEA Induced Cell Cycle Arrest Through Cell Cycle Regulators Expression

#### 3.3.1 Cyclin B1

Among the known cyclins, cyclin B1 plays a regulatory subunit for CDK1, which is essential for the transition from G2 phase to mitosis. Increasing evidence demonstrates that cyclin B deregulation could contribute to the chromosomal instability observed in human cancer (Jin et al., 1998; Park et al., 2000; Yin et al., 2001). Overexpression of cyclin B1 is observed in various human tumors (Wang et al., 1997; Park et al., 2000; Soria et al., 2000; Hassan et al., 2002); and is related to resistance to radiotherapy (Hassan et al., 2002)and other adjuvant therapy (Suzuki et al., 2007). Some researchers regard the depletion of cyclin B1 as a strategy for antiproliferative therapy. Yuan et al. (2004) evaluated the impact of small interfering RNAs (siRNAs) targeted to cyclin B1 on different human tumor cell lines. Cyclin B1 siRNAs reduced the protein level of cyclin B1 and efficiently reduced the kinase activity of Cdc2/cyclin B1. siRNA-treated cells were arrested in G2/M phase. Androic et al. (2008) studied the effect of small interfering RNA (siRNA) on different gynecological cancer cell lines knockdown of cyclin B1 inhibits proliferation *in vitro* as well as *in vivo*. Therefore, specific cyclin B1 targeting is an attractive strategy. In this study demonstrated that CDDO-TFEA induced cell cycle arrest in the GBM cells. Figure 7 displays the results of the Western blotting analysis of cell proteins extracted from the treated cells and cyclin B1 was significantly downregulated after CDDO-TFEA treatment. Therefore, the above results suggest that CDDO-TFEA induced cell cycle arrest through cyclin B1 downregulation in the GBM cells.

#### 3.3.2 Cyclin A2

Cyclin A2, as a cell cycle regulator, is commonly associated to dividing cells and is usually taken as a marker of cell proliferation (Yam et al., 2002). Cyclin A2 binds to and activates cyclin-dependent kinases, CDK1 and CDK2 (Kanakkanthara et al., 2016). Cyclin A2 expression is induced when entering S phase (Erlandsson et al., 2000), persists in S and G2 phases (Den elzen and Pines, 2001), and is degraded when entering mitosis (Geley et al., 2001). Deregulation of cyclin A2 has been reported in a variety of cancers (Malumbres and Barbacid, 2009; Gopinathan et al., 2014), and is related to chromosomal instability and tumor proliferation (Bukholm et al., 2001). In addition, cyclin A2 expression was found to be a potential prognostic biomarker (Chao et al., 1998; Chen et al., 2004; Poikonen et al., 2005). In this experiment, the relative intensity of cyclin A2 was significantly downregulated after CDDO-TFEA treatment. Based on the above results, it is implied that when CDDO treatment is used, it will inhibit cyclin A2 expression, thereby inhibiting or reducing the binding to CDK, causing cells to fail to progress the S and G phases, and ultimately leading to cell cycle arrest.

#### 3.3.3 Cyclin B1/CDK1 Complexes

Cancer cells frequently exhibit unscheduled division. The cell cycle is controlled by a family of cyclin dependent kinases (CDKs) and regulatory subunits cyclins (Krek and Nigg, 1991). As their name implies, the activities of these kinases are modulated by cyclins (Hartwell and Weinert, 1989). The cyclin B1/CDK1 complex was demonstrated to regulate cells’ entry into mitosis (Nurse, 1990). Numerous proteins are phosphorylated by the cyclin B1/CDK1 complex prior to mitotic entry. Through its cytoplasmic, nuclear, and centrosomal localization, cyclin B1/CDK1 is able to synchronize various events in mitosis, including nuclear envelope breakdown and centrosome separation (Takizawa and Morgan, 2000). Regulation of the mitotic events is linked to the control of the activity of the cyclin B1/CDK1 complex to make cells enter mitosis, arrest at the G2 phase, or skip mitosis (Wang Z. et al., 2014). We demonstrated herein that treatment with CDDO-TFEA led to an incremental increase in the population of cells in the G2/M phase, indicative of a delay in the G2/M checkpoint. In addition, cell failure to pass the G2 checkpoint leads to cell cycle arrest in the G2/M phase in MPM-2 staining. Furthermore, cyclin A2, cyclin B1, and CDK1 were significantly downregulated in the GBM8401 cells in Figure 7. As shown in Figure 8, a significant reduction in cyclin B1/CDK1 complexes was observed. Taken together, these suggests that regarding cell failure to pass the G2 checkpoint leads to cell cycle arrest in the G2/M phase may occur through cyclin B1, CDK1, and cyclin B1/CDK1 complexes downregulation after CDDO treatment.

### 3.4 CDDO-TFEA Induced Cell Cycle Arrest Through Checkpoint Kinases Upregulation

The activated cell cycle checkpoints delay cell cycle progress to promote DNA repair. The checkpoints can also eliminate harmful damaged cells by inducing cell death, thereby protecting the organism from cancer (Zhou and Elledge, 2000). Checkpoint kinases 1 and 2 (CHK1 and CHK2) play important signal transducer roles in cell cycle checkpoints. The task of CHK1 and CHK2 are to transmit checkpoint signals from the checkpoint kinases of the phosphatidylinositol 3 kinase family such ATM and ATR (Kastan and Lim, 2000; Abraham, 2001; Beishline and Azizkhan-Clifford, 2014), which phosphorylate and activate CHK1 and CHK2. CHK2 is a stable protein expressed throughout the cell cycle. In contrast, the unstable CHK1 protein is mainly limited to the S and G2 phases (Lukas et al., 2001). According to different conditions, different pathways are involved, ATM-CHK2-p53 pathway controls G1 checkpoint or ATR-CHK1-Wee1 pathway controls S and G2/M checkpoints (Shiloh and Ziv, 2013; Lin et al., 2017; Smith et al., 2020). Alterations of CHK1 and CHK2 expression are suggested contributors to the development of both hereditary and sporadic human cancer (Stawinska et al., 2008). The relative intensities of human checkpoints kinase such as CHK1, CHK2 and p-CHK2. As Figure 7 shown, the relative intensity of CHK1 and the ratio of p-CHK2/CHK2 were significantly unregulated expression. Based on the above results, it is implied that CDDO-TFEA induce the Checkpoint kinases, it will induce CHK1 and the ratio of p-CHK2/CHK2 expression, thereby inhibiting the CDK, causing cells to fail to cell progression, and ultimately leading to cell cycle arrest or inducing cell death.

### 3.5 CDDO-TFEA Induces Apoptosis and G2/M Arrest in Glioblastoma Multiforme

**FIGURE 9:**
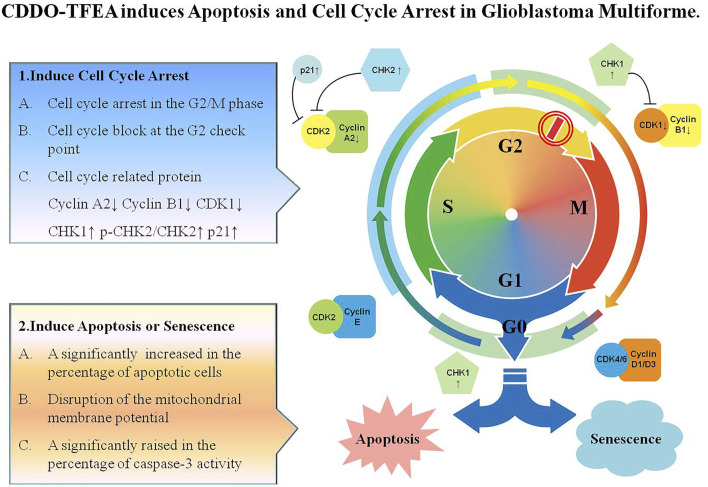
CDDO-TFEA induces apoptosis and cell cycle arrest in Glioblastoma Multiforme. CDDO-TFEA may not only induce cell cycle arrest but also induce apoptosis in established GBM cells. G1 phase: Growth 1 phase. S phase: Synthesis phase. G2 phase: Growth 2 phase. M phase: mitosis. CDK1: Cyclin Depandant Kinase 1. CDK2: Cyclin Depandant Kinase 2. CHK1: Checkpoint Kinase 1. CHK2: Checkpoint Kinase 2.

Leading to cell cycle arrest and inducing cell apoptosis are the two main strategies for suppressing cancerous tumors. As shown in Figure 9, CDDO-TFEA induces apoptosis and G2/M arrest in GBM. First, CDDO-TFEA leads to cell cycle arrest. Treatment with CDDO-TFEA, the cell division process is blocked at the G2 checkpoint, resulting in an increase in tumor cells in the G2/M phase cells, and cell cycle arrest. In addition, CDDO-TFEA induces cell apoptosis or senescence. When the cell cycle is arrested, undivided cells are induced by drugs to induce cell apoptosis or senescence, thereby further achieving anti-cancer and anti-proliferation effects. Therefore, CDDO-TFEA, a novel and potential anticancer drug, induces cell apoptosis and induces cycle arrest in GBM.

## 4 Conclusion

CDDO-TFEA significantly inhibited the proliferation of GBM8401 cells and induced apoptosis therein. Specifically, cell failure to pass the G2 checkpoint leads to cell cycle arrest in the G2/M phase by inhibiting the expression of cyclin B1, CDK1, and cyclin B1/CDK1 complexes when exposure to CDDO-TFEA. The findings suggest that CDDO-TFEA can inhibit cell cycle progression and induce apoptosis in GBM cells *in vitro*, possibly though its inhibition of Cyclin B1, CDK1 expression, and Cyclin B1/CDK1 association and the promotion of CHK1 and p-CHK2 expression. The CDDO-TFEA, a novel and potential anticancer drug, induces cell apoptosis and induces cycle arrest in GBM.

## 5 Materials and Methods

### 5.1 Materials

2-cyano-3,12-dioxo-N-(2,2,2-trifluoroethyl)-oleana-1,9(11)-dien-28-amide, CDDO-TFEA, was purchased from Cayman Chemical, DMSO (dimethyl sulfoxide) and PrestoBlue™ Cell Viability Reagent (PB) were purchased from ThermoFisher (USA). Cell culture medium (DMEM), fetal bovine serum, antibiotics, sodium pyruvate, trypsin, and phosphate-buffered saline (PBS) were obtained from Gibco, BRL (Grand Island, NY), and polyvinylidene fluoride membrane (PVDF) (Millipore), and molecular weight markers from Bio-Rad (USA). All other reagents and compounds were of analytical grade.

#### 5.1.1 Cell Culture

Human brain malignant glioma GBM 8401 cells were obtained from Bioresource Collection and Research Center (BCRC, Hsinchu, Taiwan). All cell lines were incubated in an atmosphere containing 5% CO_2_ at 37°C. GBM8401 cells were cultured in an RPMI1640 medium with supplemental 10% fetal bovine serum (FBS) and U87 MG cells in modified Eagle’s medium (MEM) with supplemental 10% FBS (Huang et al., 2010).

### 5.2 Cell Viability

A density of 5,000 cells was suspended in a culture medium containing 10% FBS and placed in a 96-well plate (0.1 ml of medium per each well) and incubated in an atmosphere containing 5% CO_2_, saturated humidity, and 37°C for 24 h. The cells were added with 0, 1, 1.5, 2 μM CDDO-TFEA and incubated with PrestoBlue™ Cell Viability Reagent for 10 min. The reaction was measured at 570 nm using a multiwell plate reader (lQUANT; BioTek Instruments, Inc., USA) (Huang et al., 2012). When there were no cells, we subtracted the background absorbance of the medium. All samples were assayed at least in triplicate, and the mean was calculated for each experiment. Results were expressed as a percent of control, control being 100%. Each experiment was performed in triplicate with a mean (+/-SEM) used to express results.

### 5.3 Cell Cycle Analysis

The GBM8401 cells were plated in 6-well plates (1 × 10^6^) in a cell incubator and cultured overnight. The next day, the cells were centrifuged in a 10 ml centrifuge tube and the supernatant was collected. They were washed two times in PBS and added with 1x Trypsin. They were then placed in a 37°C oven for 1–2 min. Once the cells fell off, they were collected in a centrifuge tube run at 2,500 rpm for 5 min to remove the supernatant. Then, 1 ml of PBS was added to wash the remaining culture solution and the cells were centrifuged again at 2,500 rpm for 5 min. 500 µl of PBS was added to break up the cell pallet and then 500 µl of 70% ethanol was slowly added to the cells for fixation. They were placed in a refrigerator and left there overnight. The next day those cells were centrifuged at 2,500 rpm for 5 min, and the supernatant containing Ethanol was removed. The cells were washed in 1 ml PBS. 5 μl of RNAse A 100 mg/ml was added to PBS and placed in an oven at 37°C.

To facilitate cell cycle analysis, a fluorescent nucleic acid dye Propidium Iodide (PI) was used to identify the proportion of cells in each of the three inter-phase stages. After a 30-min reaction time, 20 µl of propidium iodide 2 mg/ml (final concentration 40 μg/ml) was added and the cells were placed an oven at 37°C for 15 min. The cells were treated with CDDO-TFEA for 24 h followed by harvesting and fixing in 1 ml of ice-cold ethanol (70%) at −20°C for at least 8 h. DNA was stained with PI/RNaseA staining buffer, and the cell cycle was analyzed using a FACSCalibur flow cytometer. Data were interpreted using WinMDI 2.9 software (Huang et al., 2012).

### 5.4 Apoptosis Measurement

The cells were cultured in 6 well culture plates (Orange Scientific, EU). After exposure to CDDO-TFEA for 4 h, the cells were harvested by centrifugation, resuspended in, and incubated with 1x annexin-binding buffer containing 5 lL of annexin V-FITC and 1 lL of Propidium Iodide (PI) (100 mg/ml), and incubated at room temperature for 15 min. The stained cells were analyzed on a FACSCalibur flow cytometer (BD Pharmingen)using WinMDI 2.9 free software (BD Pharmingen) (Huang et al., 2010).

### 5.5 Evaluation of Mitochondrial Membrane Potential

The cells were seeded into 24-well plates (Orange, United Kingdom). Following treatment with CDDO-TFEA for 6 h, we added 10 μg/ml JC-1 (Sigma, USA) to the culture medium at 50 µl/well, which was then incubated at 37°C for 20 min for mitochondrial staining. After being washed twice with warm PBS, the cells were fixed with 2% paraformaldehyde and detected by FACS Calibur flow cytometer (JC-1). Data were analyzed using WinMDI 2.9. JC-1 was also detected by using fluorescence microscopy (Olympus CKX41 and U-RFLT 50, Japan).

### 5.6 Western Blotting

All samples were lysed in 200 μl of lysis buffer. A total of 50–75 μg of protein per sample were loaded onto 10–12% sodium dodecyl sulfate-polyacrylamide gel electrophoresis membranes for electrophoretic separation and then transferred to PVDF membranes and subjected to electrophoresis at 50 V for 4 h. After blocking overnight with Odyssey blocking buffer (USA), the membranes were incubated with primary antibodies [Cyclin A2 (1:1,000; proteintech; 18202-1-AP), Cyclin B1 (1:1,000; proteintech; 55004-1-AP), CDK1 (1:1,000; cell signaling; E1Z6R), NRF2 (1:1,000; proteintech; 16396-1-AP), CHK2 (1:1,000; abgent.com; AP4999a), p-CHK2 (1:1,000; abgent; AP50241), CHK1 (1:1,000; proteintech; 22018-1-AP), p21 (1:1,000; Cell Signaling; #2947), and β-actin (1:20,000; Sigma; A5441)] for 2 h at room temperature. Subsequently, the membranes were washed several times and then incubated with a corresponding secondary antibody (IRDye Li-COR, USA) at a dilution of1:20,000 for 30–45 min. Antigens were then visualized using a near-infrared fluorescence imaging system (Odyssey LICOR,USA), and the data were interpreted using the Odyssey2.1 software or a chemiluminescence detection kit (ECL; Amersham Corp., Arlington Heights, IL, USA).

### 5.7 Mitotic Index Analysis

Mitotic index was assessed based on MPM-2 (anti-phospho-Ser/Thr-Pro) expression (Cheng et al., 2019). After treatment with CDDOTFEA, cells were harvested and fixed in 70% ethanol overnight. The cells were then washed and suspended in 100 µl of IFA-Tx buffer (4% FCS, 150 nM NaCl, 10 nM HEPES, 0.1% sodiumazide, and 0.1% Triton X-100) with the MPM-2 antibody at room temperature for 1 h. The cells were then washed and resuspended in IFA-Tx buffer with a rabbit anti-mouse FITC-conjugated secondary antibody (1:50 dilution; Serotec) for 1 h at room temperature in the dark. Finally, the cells were washed and resuspended in 500 µl of PBS with 20 μg/ml Propidium Iodide (PI) (Sigma) for 30 min in the dark. MPM-2 expression was measured by FACSCalibur flow cytometer. Data were analyzed using WinMDI 2.9.

### 5.8 Co-Immunoprecipitation

Co-IP is an effective means of quantifying protein-protein interactions in cells. Briefly, after incubation at room temperature overnight, 500 mg of cellular proteins were labeled using anti-CDK1 antibody. The protein–antibody immunoprecipitates were collected using protein A/G plus-agarose beads (SC-2003 Santa Cruz BioTechnology). Following the final wash, the samples were boiled and centrifuged to transform the agarose beads into pellets. Finally, cyclin B1 proteins were performed using western blot analysis Antigens were visualized and data were analyzed using Odyssey 2.1 software (Cheng et al., 2019).

### 5.9 Data Analysis

Data are expressed as the mean ± standard error of the mean of at least three independent experiments. Student’st-test or one-way analysis of variance with Scheffe’s posthoc test was used for statistical analysis. A *p* value of < 0.05 was considered statistically significant.

## Data Availability

The original contributions presented in the study are included in the article/Supplementary Material, further inquiries can be directed to the corresponding author.

## Abbreviations

ARE, antioxidant response element; ATM, ataxia-telangiectasia mutated; ATR, ATM and RAD3-related; CDDO, 2-cyano-3-,12-dioxoolean-1,9-dien-28-oic acid; CDDO-Im, CDDO imidazolide; CDDO-Me, CDDO methyl ester; CDDO-TFEA, CDDO trifluoroethyl amide; CDK1, cyclin depandant Kinase 1; CDK2, cyclin depandant kinase 2; CHK1, checkpoint kinase 1; CHK2, checkpoint kinase 2; GBM, glioblastoma multiforme; Keap1, Kelch-like ECH-associated protein 1; Nrf2, nuclear factor erythroid 2-related factor 2; RNS, reactive nitrogen species; PB, peripheral blood; PI, propidium Iodide; WHO, world health organization.
