# Supplementary material for: Induction of Mitosis Delay and Apoptosis by CDDO-TFEA in Glioblastoma Multiforme
Source: Front Pharmacol. 2021 Nov 8;12:756228. doi: 10.3389/fphar.2021.756228 (PMC8630575; doi:10.3389/fphar.2021.756228)

# Supplement data

**1. Antibody:**

**Cyclin A2 (1:1000; proteintech; 18202-1-AP)**
**Cyclin B1 (1:1000; proteintech; 55004-1-AP)**
**CDK1 (1:1000;cell signaling; E1Z6R )**
**NRF2 ( 1:1000; proteintech; 16396-1-AP)**
**CHK2 ( 1:1000; abgent.com; AP4999a)**
**p-CHk2 ( 1:1000; abgent; AP50241)**
**CHK1 (1:1000; proteintech; 22018-1-AP)**
**p21 (1:1000; Cell Signaling; #2947)β-actin (1:20000; Sigma; A5441)**

**2. Image assay software: ImageJ, NIH.**

**3. Analysis software: SPSS**

# Supplement data

## Raw data

## Figure 1 Prestoblue

| | | | | |
|---|---|---|---|---|
| | 0 | 0.492422 | 0.294885 | 0.671844 |
| | 0 | 1 | 1.5 | 2 |
| 48 hr | | | | |
| | 100 | 15.42 | 3.20 | 0.87 |
| | 0 | 0.167555 | 0.229441 | 0.339743 |
| | 0 | 1 | 1.5 | 2 |
| 72 hr | | | | |
| | 100 | 10.78 | 1.10 | 0.51 |
| | 0 | 0.249559 | 0.080048 | 0.087969 |
| | 0 | 1 | 1.5 | 2 |

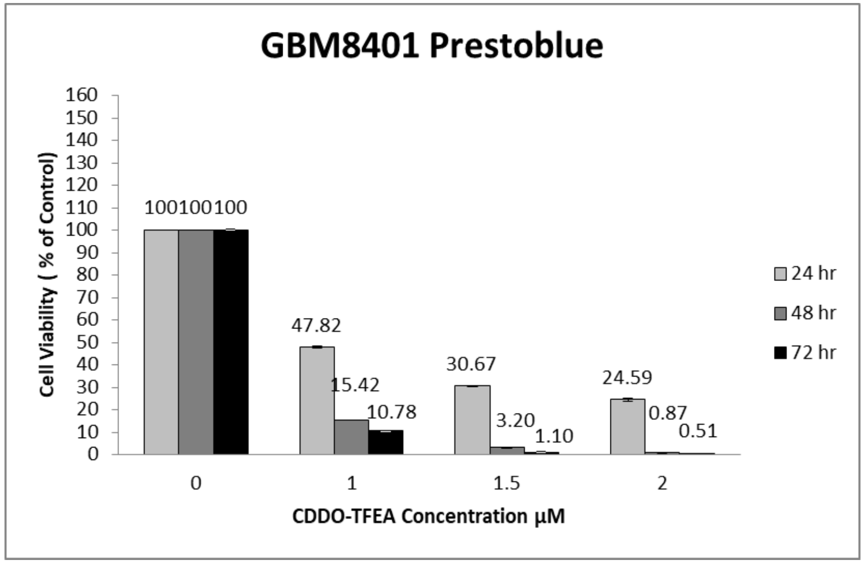


| | |
|---|---|
| 24 h | y = -24.339x + 111.61 |
| | R2 = 0.841 |
| 48 h | y = -30.959x + 107.27 |
| | R2 = 0.7175 |
| 72h | y = -30.813x + 105.13 |
| | R2 = 0.6821 |

# Supplement data

## Raw data

## Figure 2

N1

| | Control | Necrosis | Total apoptosis |
|---|---|---|---|
| DMSO | 98.54 | 0.02 | 1.44 |
| 0 | 97.62 | 0.03 | 2.35 |
| 1 | 98.13 | 0.07 | 1.8 |
| 1.5 | 97.16 | 0.05 | 2.79 |
| 2 | 96.61 | 0.06 | 3.33 |

N2

| | Control | Necrosis | Total apoptosis |
|---|---|---|---|
| DMSO | 98.11 | 0.03 | 1.86 |
| 0 | 98.16 | 0.09 | 1.75 |
| 1 | 97.36 | 0.13 | 2.51 |
| 1.5 | 95.14 | 0.09 | 4.77 |
| 2 | 93.96 | 0.03 | 6.01 |

N3

| | Control | Necrosis | Total apoptosis |
|---|---|---|---|
| DMSO | 98.4 | 0.02 | 1.58 |
| 0 | 97.62 | 0.03 | 2.35 |
| 1 | 96.7 | 0.05 | 3.25 |
| 1.5 | 95.03 | 0.09 | 4.88 |
| 2 | 92.23 | 0.02 | 7.75 |

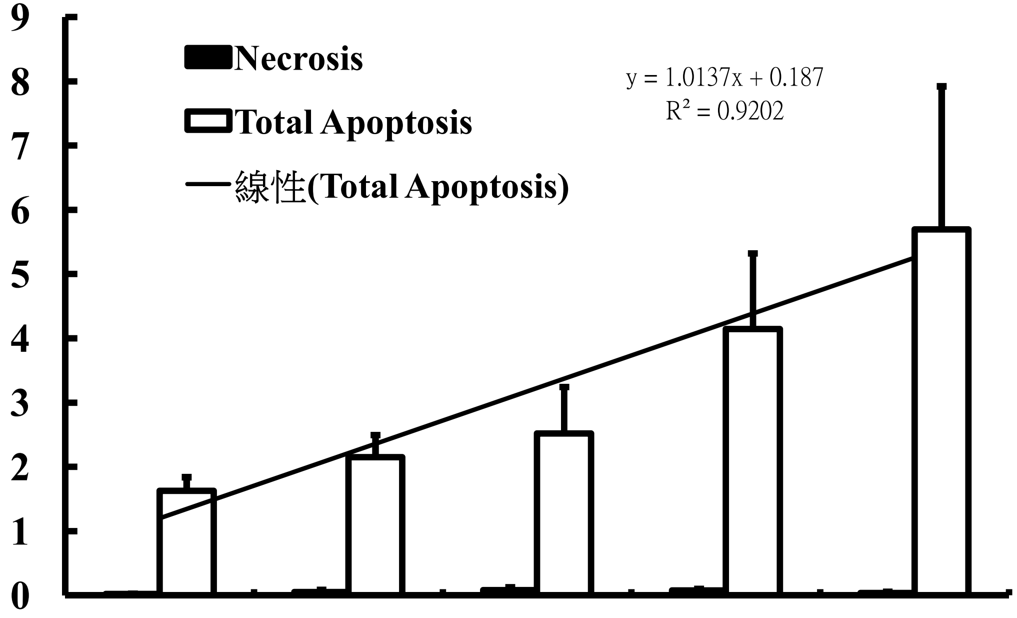

# Supplement data

## Raw data
## Figure 3 JC-1

N1

| | Agreggates (Red) | Monomers (Green) |
|---|---|---|
| 0 | 80.35 | 19.65 |
| DMSO | 86.24 | 13.76 |
| 1 | 89.78 | 10.22 |
| 1.5 | 70.2 | 29.8 |
| 2 | 23.17 | 76.83 |

N2

| | Agreggates (Red) | Monomers (Green) |
|---|---|---|
| 0 | 82.07 | 17.93 |
| DMSO | 95.91 | 4.09 |
| 1 | 87.32 | 12.68 |
| 1.5 | 76.68 | 23.32 |
| 2 | 50.61 | 49.39 |

N3

| | Agreggates (Red) | Monomers (Green) |
|---|---|---|
| 0 | 91.13 | 8.87 |
| DMSO | 85.09 | 14.91 |
| 1 | 95.06 | 4.94 |
| 1.5 | 88.58 | 11.42 |
| 2 | 77.18 | 22.82 |

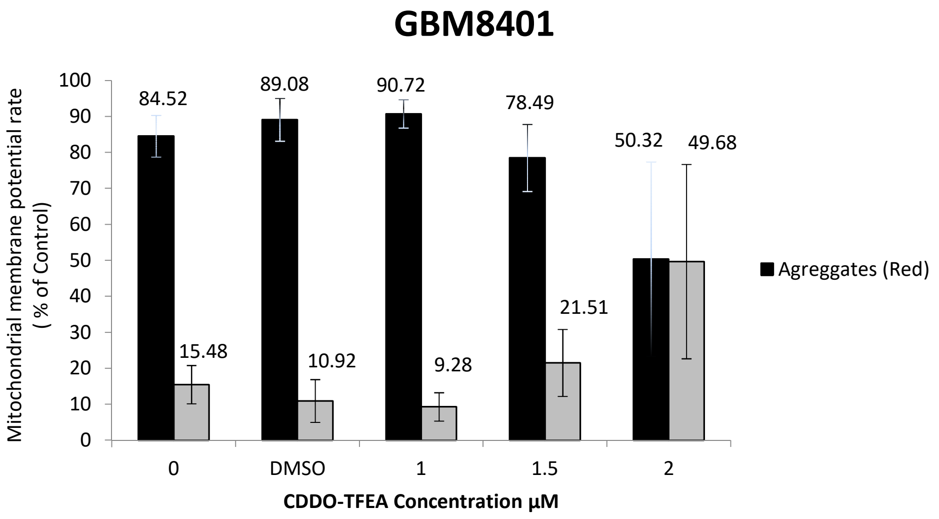

# Supplement data

## Raw data
## Figure 4 Caspase 3

| N1 | ctrl | active |
|---|---|---|
| 0 μM | 99.86 | 0.14 |
| 1 μM | 97.45 | 2.55 |
| 1.5 μM | 90.29 | 9.71 |
| 2 μM | 91.23 | 8.77 |

| N2 | ctrl | active |
|---|---|---|
| 0 μM | 99.74 | 0.26 |
| 1 μM | 95.95 | 4.05 |
| 1.5 μM | 91.41 | 8.59 |
| 2 μM | 92.05 | 7.95 |

| N3 | ctrl | active |
|---|---|---|
| 0 μM | 99.87 | 0.13 |
| 1 μM | 97.02 | 2.98 |
| 1.5 μM | 93.24 | 6.76 |
| 2 μM | 92.72 | 7.28 |

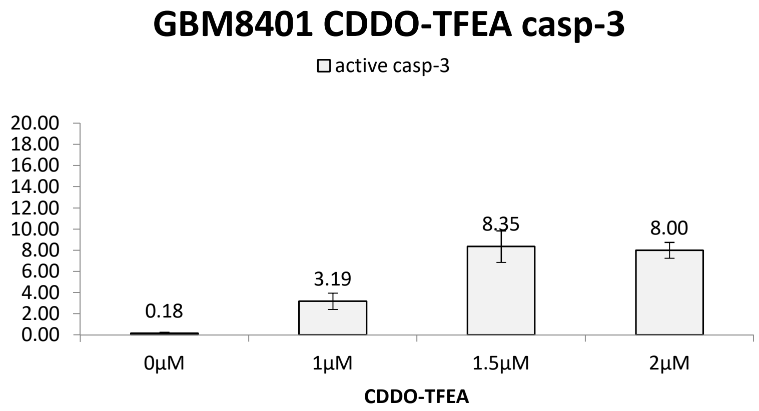

# Supplement data

**Raw data**
**Figure 5 PI**

| SubG1 | G1 | S | G2/M |
|---|---|---|---|
| 0.09 | 68.60 | 13.83 | 17.82 |
| 0.28 | 57.59 | 4.12 | 38.24 |
| 0.12 | 54.19 | 15.41 | 30.92 |
| 0.07 | 59.07 | 14.28 | 27.32 |

| subG1 | G1 | S | G2/M |
|---|---|---|---|
| 0.05 | 1.64 | 2.06 | 0.59 |
| 0.05 | 1.63 | 1.14 | 1.10 |
| 0.10 | 1.63 | 1.35 | 2.32 |
| 0.10 | 1.88 | 1.67 | 0.47 |

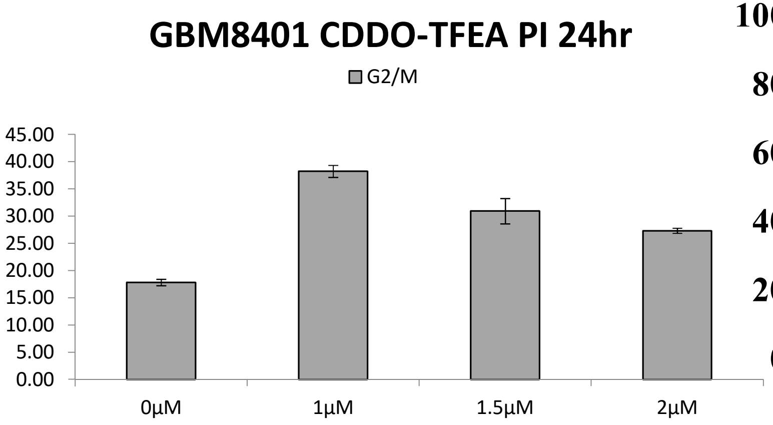


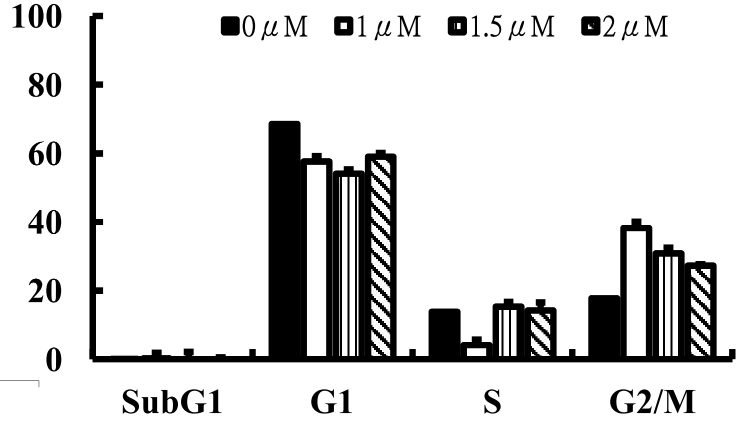

# Supplement data

## Raw data
## Figure 6 MPM2

| N1 | active | unactive |
|---|---|---|
| 0 μM | 1.27 | 98.8 |
| 1 μM | 41.55 | 59.25 |
| 1.5 μM | 51.88 | 48.78 |
| 2 μM | 63 | 37.84 |
| Nocod. | 50.63 | 50.14 |

| N2 | active | unactive |
|---|---|---|
| 0 μM | 3.37 | 96.71 |
| 1 μM | 36.52 | 64.15 |
| 1.5 μM | 53.47 | 47.33 |
| 2 μM | 64.01 | 36.71 |
| Nocod. | 50.02 | 50.63 |

| N3 | active | unactive |
|---|---|---|
| 0 μM | 9.83 | 90.36 |
| 1 μM | 55.11 | 45.62 |
| 1.5 μM | 48.73 | 51.93 |
| 2 μM | 46.84 | 54.02 |
| Nocod. | 50.02 | 50.69 |

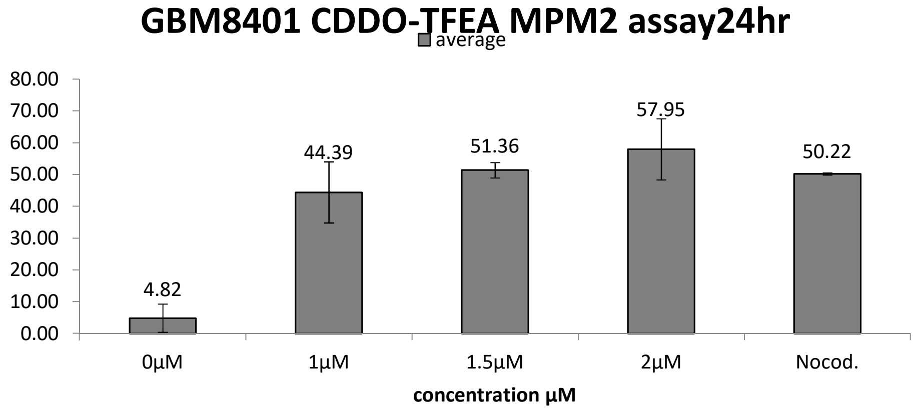

# Supplement data

## Raw data
## Figure 7 WB

| CyclinA2 | | | | | | | Average | Stdev |
|---|---|---|---|---|---|---|---|---|
| 0 μM | 100 | 100 | 100 | 100 | 100 | | 100 | 0 |
| 1 μM | 24.03252 | 136.9974 | 80.89906 | 73.09071 | 95.40061 | | 83.13012 | 11.32105 |
| 1.5 μM | 18.82105 | 55.77575 | 85.87495 | 72.2236 | 87.68005 | | 81.9262 | 8.45103 |
| 2 μM | 7.370388 | 123.979 | 98.737 | 71.29918 | 98.98692 | | 89.67437 | 15.91387 |

| CyclinB1 | | | | | | | Average | Stdev |
|---|---|---|---|---|---|---|---|---|
| 100 | 100 | 100 | 100 | 100 | | | 100 | 0 |
| 75.96228 | 63.7519 | 63.64029 | 94.15495 | 92.73417 | | | 77.48279 | 14.55084 |
| 55.25341 | 49.64954 | 98.87916 | 92.73656 | 91.23462 | | | 65.37919 | 22.56608 |
| 58.53476 | 57.38114 | 134.0235 | 117.9897 | 97.59897 | | | 71.17162 | 22.89402 |

| CDK1 | | | | | | | Average | Stdev |
|---|---|---|---|---|---|---|---|---|
| 0 μM | 100 | 100 | 100 | 100 | | | 100 | 0 |
| 1 μM | 78.66307 | 75.78782 | 85.73496 | 85.09067 | | | 83.1629 | 3.910258 |
| 1.5 μM | 79.93506 | 85.24276 | 97.77343 | 82.73996 | | | 86.81615 | 9.592359 |
| 2 μM | 89.04697 | 100.0822 | 80.04194 | 69.2874 | | | 79.45877 | 9.892687 |

| NRF2 | | | | | | | Average | Stdev |
|---|---|---|---|---|---|---|---|---|
| 0 μM | 100 | 100 | 100 | 100 | 100 | | 100 | 0 |
| 1 μM | 136.8753 | 92.97196 | 112.3632 | 77.60376 | 85.16545 | | 85.24706 | 7.684427 |
| 1.5 μM | 107.3147 | 83.831 | 131.2171 | 89.98501 | 82.96513 | | 85.59371 | 3.827537 |
| 2 μM | 112.1755 | 61.9162 | 139.3053 | 49.28537 | 87.10509 | | 66.10222 | 19.25422 |

| CHK2 | | | | | | | Average | Stdev |
|---|---|---|---|---|---|---|---|---|
| 0 μM | 100 | 100 | 100 | 100 | | | 100 | 0 |
| 1 μM | 69.21576 | 111.7715 | 85.8872 | 95.46796 | | | 83.52364 | 13.28474 |
| 1.5 μM | 68.8882 | 170.8508 | 99.05891 | 97.75786 | | | 88.56832 | 17.0559 |
| 2 μM | 48.55951 | 97.64697 | 92.41285 | 109.9377 | | | 83.6367 | 31.61625 |

| p-CHK2 | | | | | | | Average | Stdev |
|---|---|---|---|---|---|---|---|---|
| 0 μM | 100 | 100 | 100 | | | | 100 | 0 |
| 1 μM | 94.39796 | 98.44212 | 98.19793 | | | | 97.01267 | 2.267696 |
| 1.5 μM | 89.31868 | 90.96146 | 110.7946 | | | | 97.0249 | 11.95314 |
| 2 μM | 96.72915 | 113.6833 | 128.7044 | | | | 113.039 | 15.99737 |

| CHK1 | | | | | | | Average | Stdev |
|---|---|---|---|---|---|---|---|---|
| 0 μM | 100 | | | | | | 100 | |
| 1 μM | 110.2073 | | | | | | 110.2073 | |
| 1.5 μM | 121.4714 | | | | | | 121.4714 | |
| 2 μM | 137.9884 | | | | | | 137.9884 | |

| P21 | | | | | | | Average | Stdev |
|---|---|---|---|---|---|---|---|---|
| 0 μM | 100 | 100 | 100 | 100 | 100 | 100 | 100 | 0 |
| 1 μM | 199.4414 | 134.5155 | 113.9827 | 107.3846 | 122.236 | 75.3773 | 114.5344 | 7.441061 |
| 1.5 μM | 283.1178 | 171.415 | 114.7717 | 107.9799 | 114.2144 | 75.79517 | 112.322 | 3.770654 |
| 2 μM | 232.387 | 171.8881 | 116.1146 | 108.4368 | 131.6624 | 76.11583 | 118.7379 | 11.83299 |

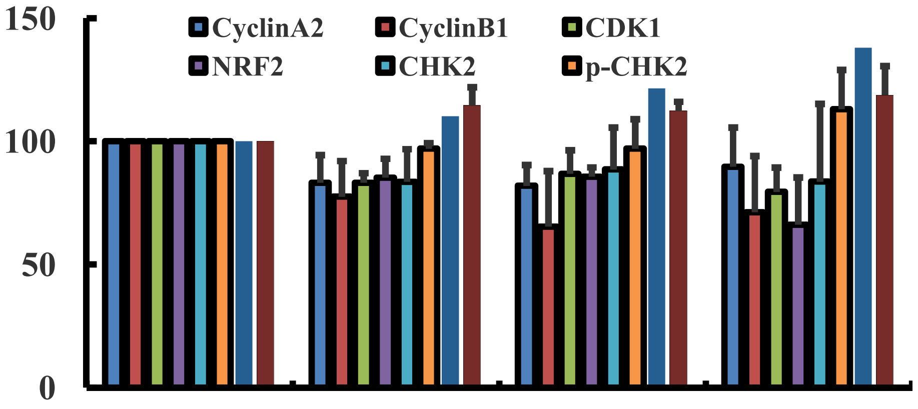

# Supplement data

## Cyclin A2/B-actin

CyclinA2

| ID/Name | Ave.Intensity | Background | Backgnd SD Mult | Integ.Intensity |
|---|---|---|---|---|
| 0-700 | 2508.69 | 0 | n/a | 60.54 |
| 1-700 | 2372.72 | 0 | n/a | 57.26 |
| 2-700 | 2126.51 | 0 | n/a | 51.31 |
| 3-700 | 2172.51 | 0 | n/a | 52.42 |

B-actin

| ID/Name | Ave.Intensity | Background | Backgnd SD Mult | Integ.Intensity |
|---|---|---|---|---|
| 0-800 | 908 | 0 | n/a | 21.91 |
| 1-800 | 900.19 | 0 | n/a | 21.72 |
| 2-800 | 877.82 | 0 | n/a | 21.18 |
| 3-800 | 794.37 | 0 | n/a | 19.17 |

| CyclinA2 | B-actin | | |
|---|---|---|---|
| 2508.69 | 908 | 2.762874 | 100 |
| 2372.72 | 900.19 | 2.635799 | 95.40061 |
| 2126.51 | 877.82 | 2.42249 | 87.68005 |
| 2172.51 | 794.37 | 2.734884 | 98.98692 |

## Cyclin A2

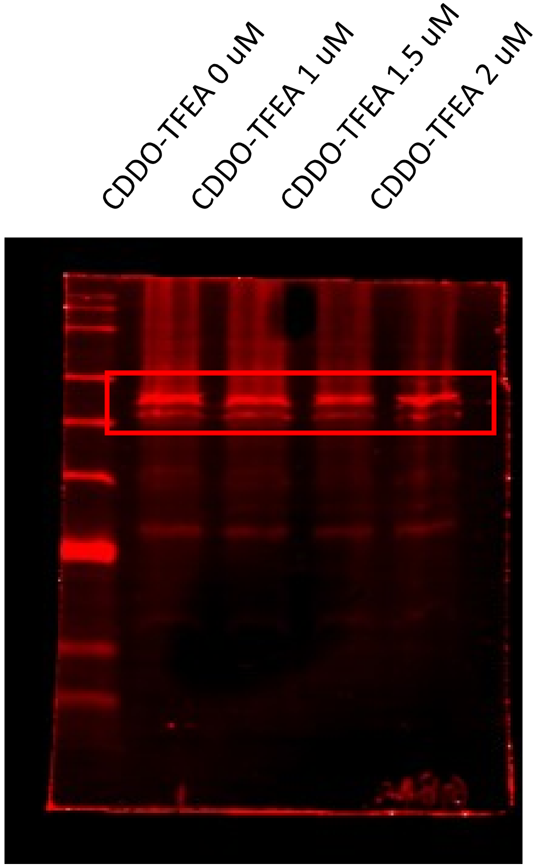


## B-actin

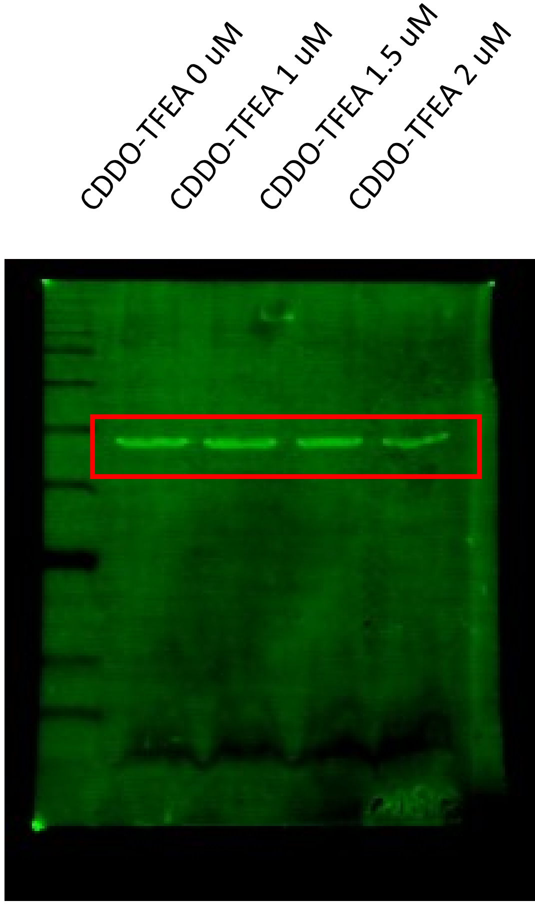

# Supplement data

## Cyclin B1/B-actin

CyclinB1

| ID/Name | Ave.Intensity | Background | Backgnd SD Mult | Integ.Intensity |
|---|---|---|---|---|
| 0-700 | 1691.02 | 0n/a | | 23.32 |
| 1-700 | 1773.65 | 0n/a | | 24.46 |
| 2-700 | 1902.75 | 0n/a | | 26.24 |
| 3-700 | 2382.46 | 0n/a | | 32.85 |

B-actin

| ID/Name | Ave.Intensity | Background | Backgnd SD Mult | Integ.Intensity |
|---|---|---|---|---|
| 0-800 | 738.31 | 0n/a | | 10.18 |
| 1-800 | 822.46 | 0n/a | | 11.34 |
| 2-800 | 895.82 | 0n/a | | 12.35 |
| 3-800 | 881.6 | 0n/a | | 12.16 |

| CyclinB1 | B-actin | % | |
|---|---|---|---|
| 1691.02 | 738.31 | 2.290393 | 100 |
| 1773.65 | 822.46 | 2.156518 | 94.2 |
| 1902.75 | 895.82 | 2.124032 | 92.7 |
| 2382.46 | 881.6 | 2.702427 | 118.0 |

## Cyclin B1

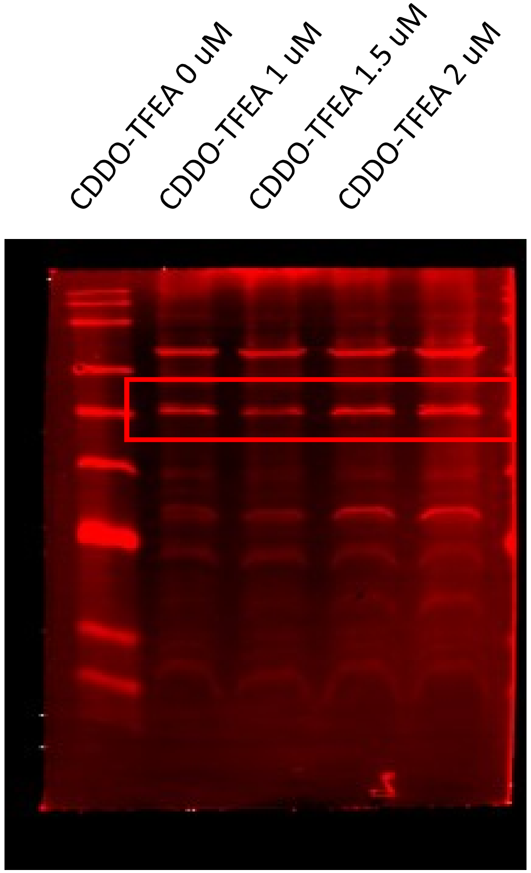


## B-actin

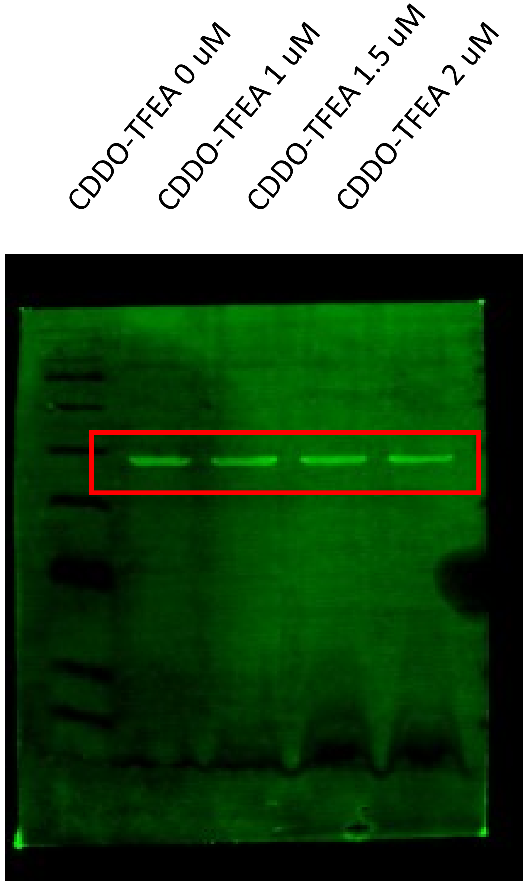

# Supplement data

## CDK1/B-actin

B-actin

| ID/Name | Ave.Intensity | Background | Backgnd SD Mult | Integ.Intensity |
|---|---|---|---|---|
| 0-800 | 483.53 | 0 | n/a | 8.26 |
| 1-800 | 487.06 | 0 | n/a | 8.33 |
| 2-800 | 470.99 | 0 | n/a | 8.05 |
| 3-800 | 449.81 | 0 | n/a | 7.69 |

CDC2

| ID/Name | Ave.Intensity | Background | Backgnd SD Mult | Integ.Intensity |
|---|---|---|---|---|
| 0-800 | 604.21 | 0 | n/a | 10.33 |
| 1-800 | 478.76 | 0 | n/a | 8.18 |
| 2-800 | 470.45 | 0 | n/a | 8.04 |
| 3-800 | 500.51 | 0 | n/a | 8.56 |

| CDC2 | B-actin | | % |
|---|---|---|---|
| 604.21 | 483.53 | 1.249581 | 100 |
| 478.76 | 487.06 | 0.982959 | 78.66307 |
| 470.45 | 470.99 | 0.998853 | 79.93506 |
| 500.51 | 449.81 | 1.112714 | 89.04697 |

## CDK1

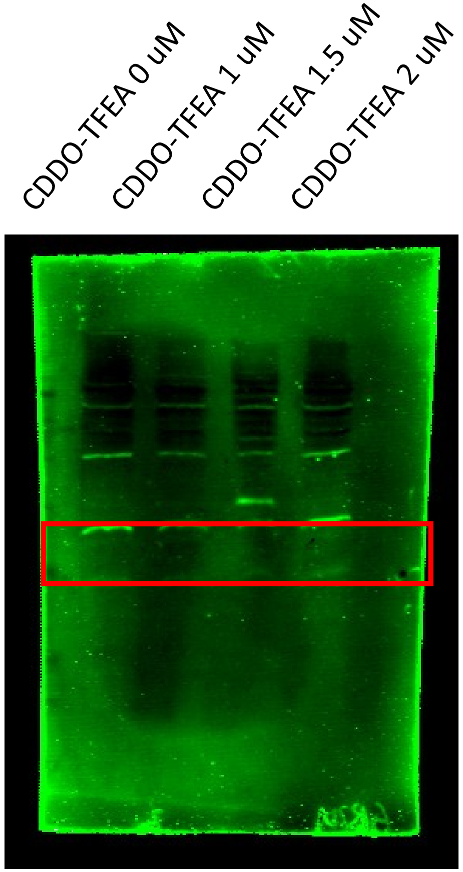


## B-actin

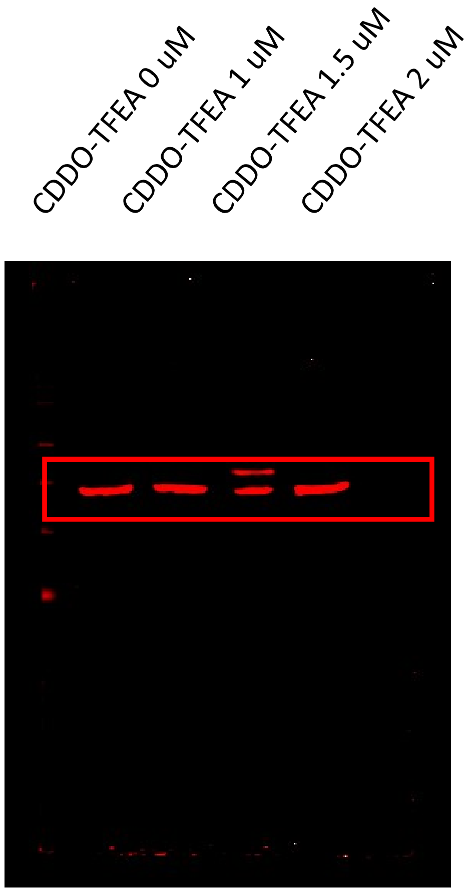

# Supplement data

## NRF2/B-actin

NRF2

| ID/Name | Ave.Intensity | Background | Backgnd SD Mult | Integ.Intensity |
|---|---|---|---|---|
| 0-700 | 1560.17 | 0n/a | | 26.89 |
| 1-700 | 1426.09 | 0n/a | | 24.58 |
| 2-700 | 1415.87 | 0n/a | | 24.4 |
| 3-700 | 1481.82 | 0n/a | | 25.54 |

B-actin

| ID/Name | Ave.Intensity | Background | Backgnd SD Mult | Integ.Intensity |
|---|---|---|---|---|
| 0-800 | 630.08 | 0n/a | | 10.86 |
| 1-800 | 676.25 | 0n/a | | 11.66 |
| 2-800 | 689.21 | 0n/a | | 11.88 |
| 3-800 | 687.03 | 0n/a | | 11.84 |

| NRF2 | B-actin | | |
|---|---|---|---|
| 1560.17 | 630.08 | 2.476146 | 100 |
| 1426.09 | 676.25 | 2.108821 | 85.16545 |
| 1415.87 | 689.21 | 2.054338 | 82.96513 |
| 1481.82 | 687.03 | 2.156849 | 87.10509 |

## NRF2

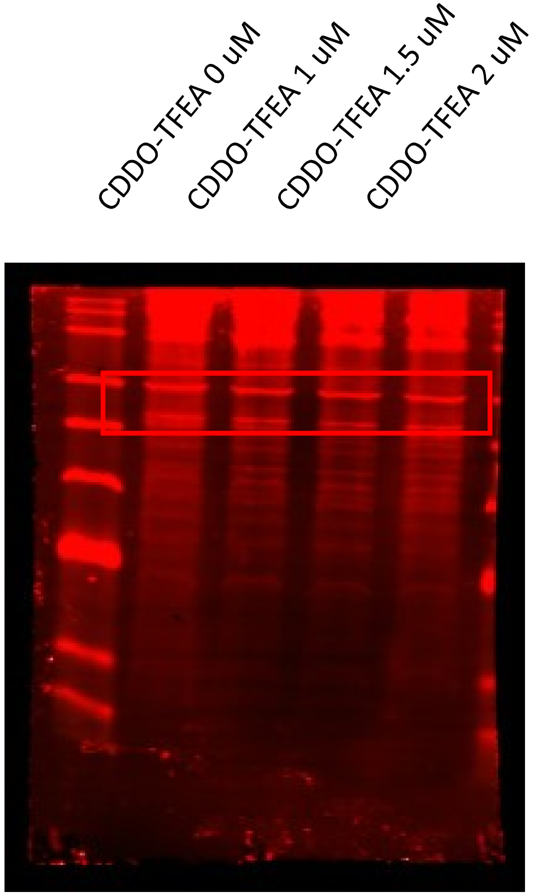


## B-actin

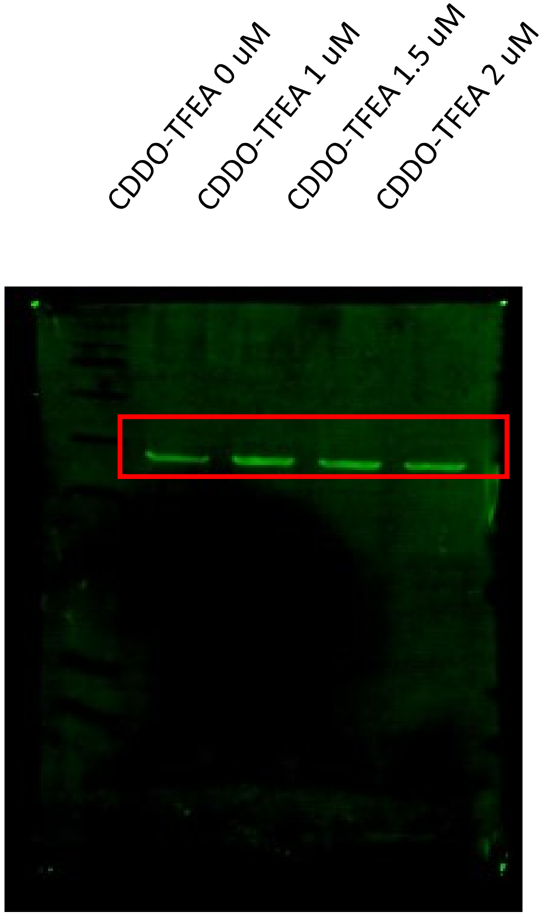

# Supplement data

## CHK2/p-CHK2/B-actin

| Ave.Inte nsity | Backgro und | Backgnd SD | Mult | Integ.Intensity |
|---|---|---|---|---|
| 417.42 | 0n/a | | | 5.01 |
| 416.44 | 0n/a | | | 5 |
| 396.1 | 0n/a | | | 4.76 |
| 349.46 | 0n/a | | | 4.2 |

| Ave.Inte nsity | Backgro und | Backgnd SD | Mult | Integ.Intensity |
|---|---|---|---|---|
| 282.37 | 0n/a | | | 3.39 |
| 268.94 | 0n/a | | | 3.23 |
| 261.94 | 0n/a | | | 3.15 |
| 259.89 | 0n/a | | | 3.12 |

| Ave.Inte nsity | Backgro und | Backgnd SD | Mult | Integ.Intensity |
|---|---|---|---|---|
| 1062.18 | 0n/a | | | 12.75 |
| 1040.59 | 0n/a | | | 12.5 |
| 1116.73 | 0n/a | | | 13.41 |
| 1144.5 | 0n/a | | | 13.74 |

## CHK2

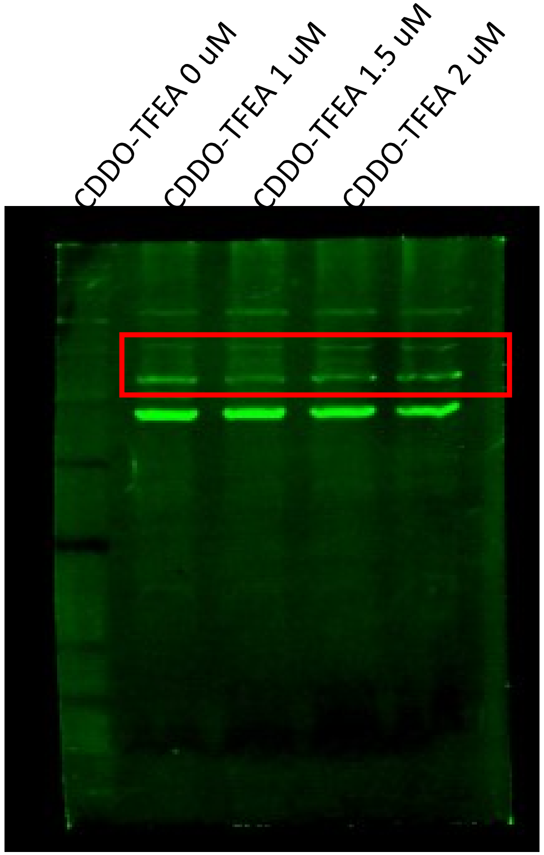


## P-CHK2

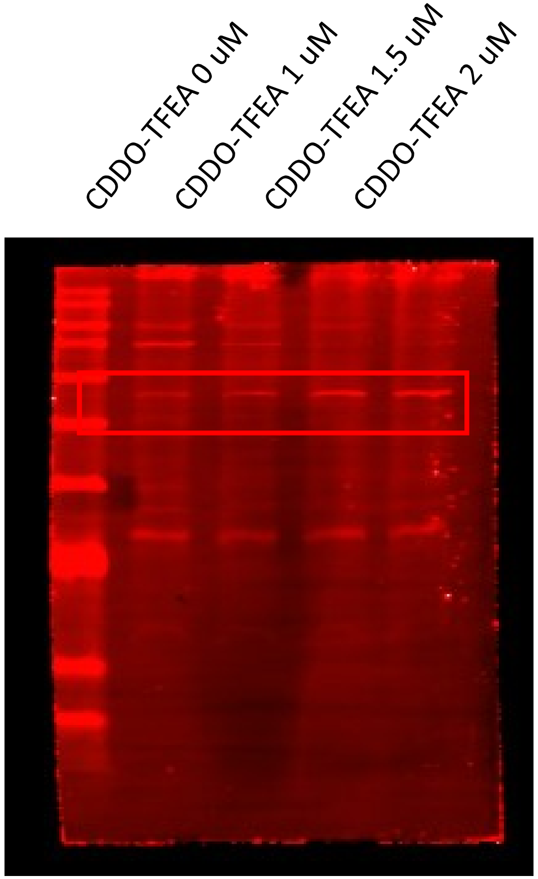


## B-actin

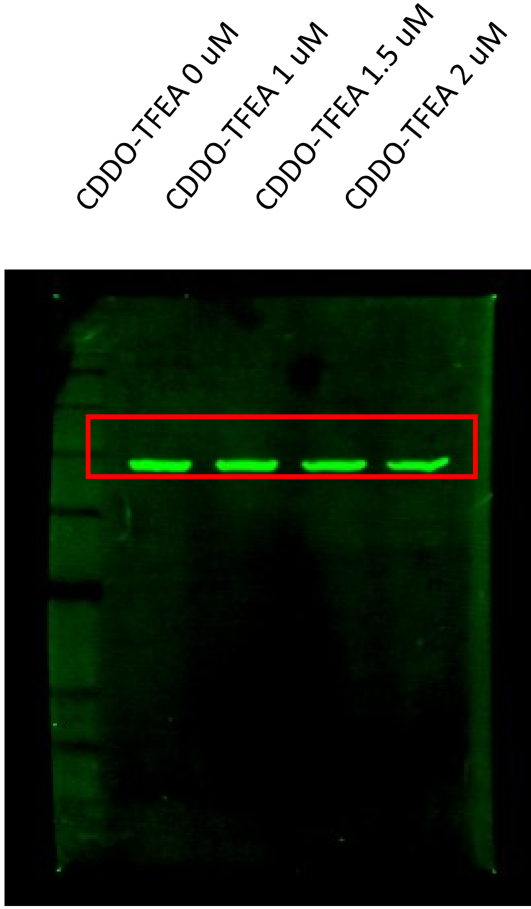

# Supplement data

## CHK1/B-actin

| b-actin | Number | Vol. % | Volume | Height | Area |
|---|---|---|---|---|---|
| 0 μM | No 1 | | 196,007,266 | 65,535 | 5,940 |
| 1 μM | No 2 | | 220,149,119 | 65,535 | 6,120 |
| 1.5 μM | No 3 | | 196,885,412 | 65,535 | 6,000 |
| 2 μM | No 4 | | 134,706,723 | 65,535 | 5,940 |

| CHK1 | Number | Vol. % | Volume | Height | Area |
|---|---|---|---|---|---|
| 0 μM | No 1 | | 82,394,099 | 65,535 | 4,895 |
| 1 μM | No 2 | | 101,988,509 | 65,535 | 5,060 |
| 1.5 μM | No 3 | | 100,533,628 | 65,535 | 5,060 |
| 2 μM | No 4 | | 78,136,817 | 65,535 | 5,005 |

| | CHk1 | b-actin | | % |
|---|---|---|---|---|
| 0 μM | 82,394,099 | 196,007,266 | 0.42036247 | 100.0 |
| 1 μM | 101,988,509 | 220,149,119 | 0.46327012 | 110.2 |
| 1.5 μM | 100,533,628 | 196,885,412 | 0.51061999 | 121.5 |
| 2 μM | 78,136,817 | 134,706,723 | 0.58005135 | 138.0 |

## CHK1

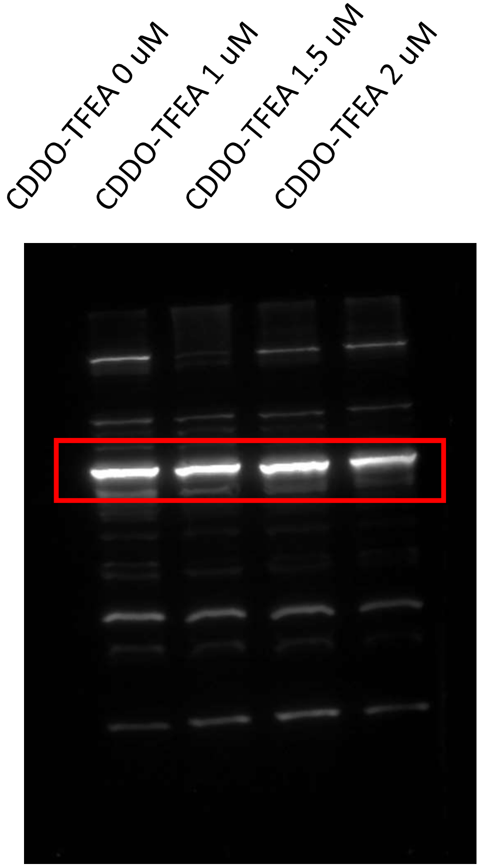


## B-actin

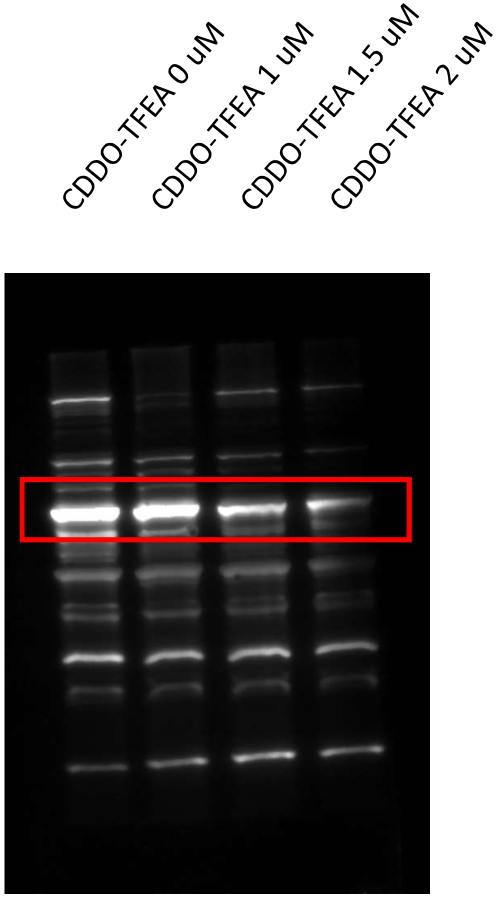

# Supplement data

## p21/B-actin

| b-actin | 20210324A | | | |
|---|---|---|---|---|
| ID/Name | Ave.Intensity | Background | Backgnd SD Mult | Integ.Intensity |
| 0-700 | 497.96 | 0 | n/a | 7.41 |
| 1-700 | 513.92 | 0 | n/a | 7.65 |
| 2-700 | 544.96 | 0 | n/a | 8.11 |
| 3-700 | 648.16 | 0 | n/a | 9.65 |
| P21 | 20210324A | | | |
| ID/Name | Ave.Intensity | Background | Backgnd SD Mult | Integ.Intensity |
| 0-800 | 321.16 | 0 | n/a | 8.76 |
| 1-800 | 355.93 | 0 | n/a | 9.71 |
| 2-800 | 379.52 | 0 | n/a | 10.36 |
| 3-800 | 453.3 | 0 | n/a | 12.37 |

| | P21 | b-actin | | % |
|---|---|---|---|---|
| 0 μM | 321.16 | 497.96 | 0.6449514 | 100 |
| 1 μM | 355.93 | 513.92 | 0.69257861 | 107.4 |
| 1.5 μM | 379.52 | 544.96 | 0.69641809 | 108.0 |
| 2 μM | 453.3 | 648.16 | 0.69936435 | 108.4 |

## p21

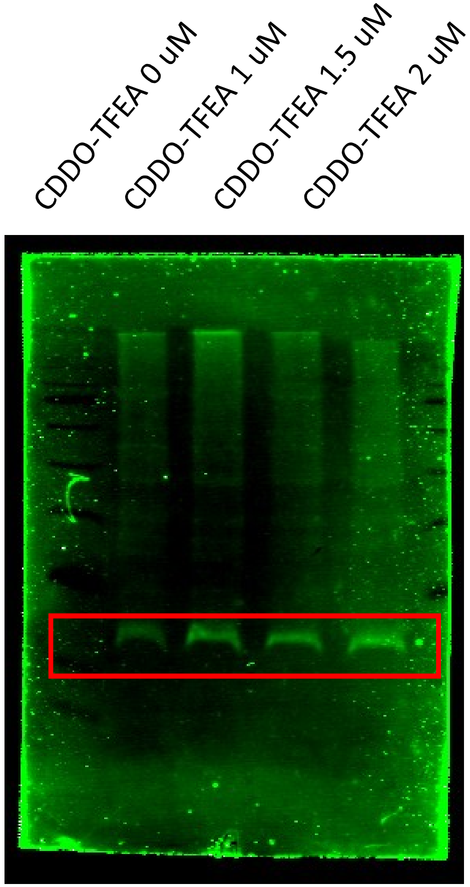


## B-actin

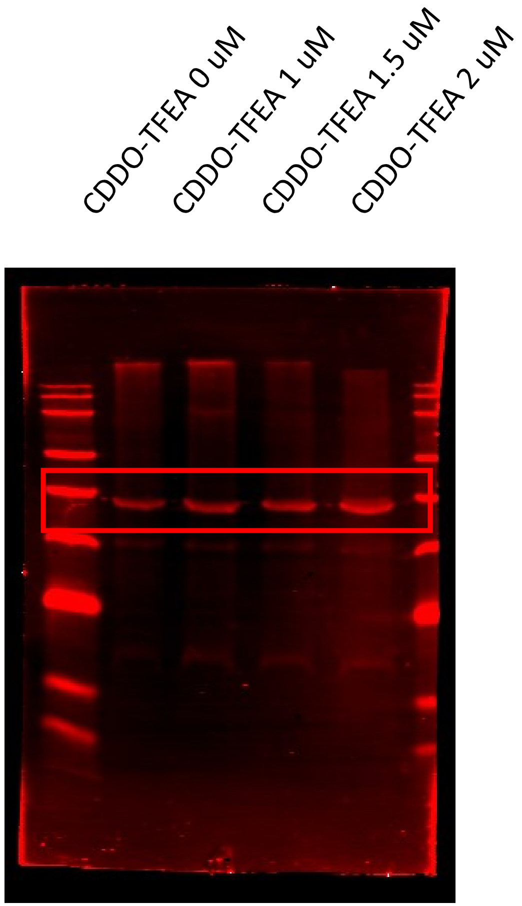

# Supplement data

## Raw data

## Figure 8 Co-immunoprecipitation (Co-IP)

CyclinB1

| ID/Name | Ave.Intensity | Background | Backgnd SD Mult | Integ.Intensity |
|---|---|---|---|---|
| 0-700 | 2381.7 | 0 | n/a | 42.69 |
| 1-700 | 2250.62 | 0 | n/a | 40.34 |
| 2-700 | 2119.57 | 0 | n/a | 38 |
| 3-700 | 2159.65 | 0 | n/a | 38.71 |
| 4-700 | 1683.6 | 0 | n/a | 30.18 |
| 5-700 | 1276.2 | 0 | n/a | 22.88 |
| 6-700 | 1589.36 | 0 | n/a | 28.49 |
| 7-700 | 1576.74 | 0 | n/a | 28.26 |

CDK1

| ID/Name | Ave.Intensity | Background | Backgnd SD Mult | Integ.Intensity |
|---|---|---|---|---|
| 0-800 | 566.79 | 0 | n/a | 11.07 |
| 1-800 | 471.71 | 0 | n/a | 9.21 |
| 2-800 | 453.73 | 0 | n/a | 8.86 |
| 3-800 | 482.39 | 0 | n/a | 9.42 |
| 4-800 | 363.3 | 0 | n/a | 7.1 |
| 5-800 | 346.38 | 0 | n/a | 6.77 |
| 6-800 | 403.5 | 0 | n/a | 7.88 |
| 7-800 | 388.22 | 0 | n/a | 7.58 |

| | CyclinB1 | CDK1 | | CyclinB1/CDK1 |
|---|---|---|---|---|
| 0 μM | 1683.6 | 363.3 | 4.634187 | 100 |
| 1 μM | 1276.2 | 346.38 | 3.684393 | 79.50463 |
| 1.5 μM | 1589.36 | 403.5 | 3.938934 | 84.99732 |
| 2 μM | 1576.74 | 388.22 | 4.06146 | 87.64127 |

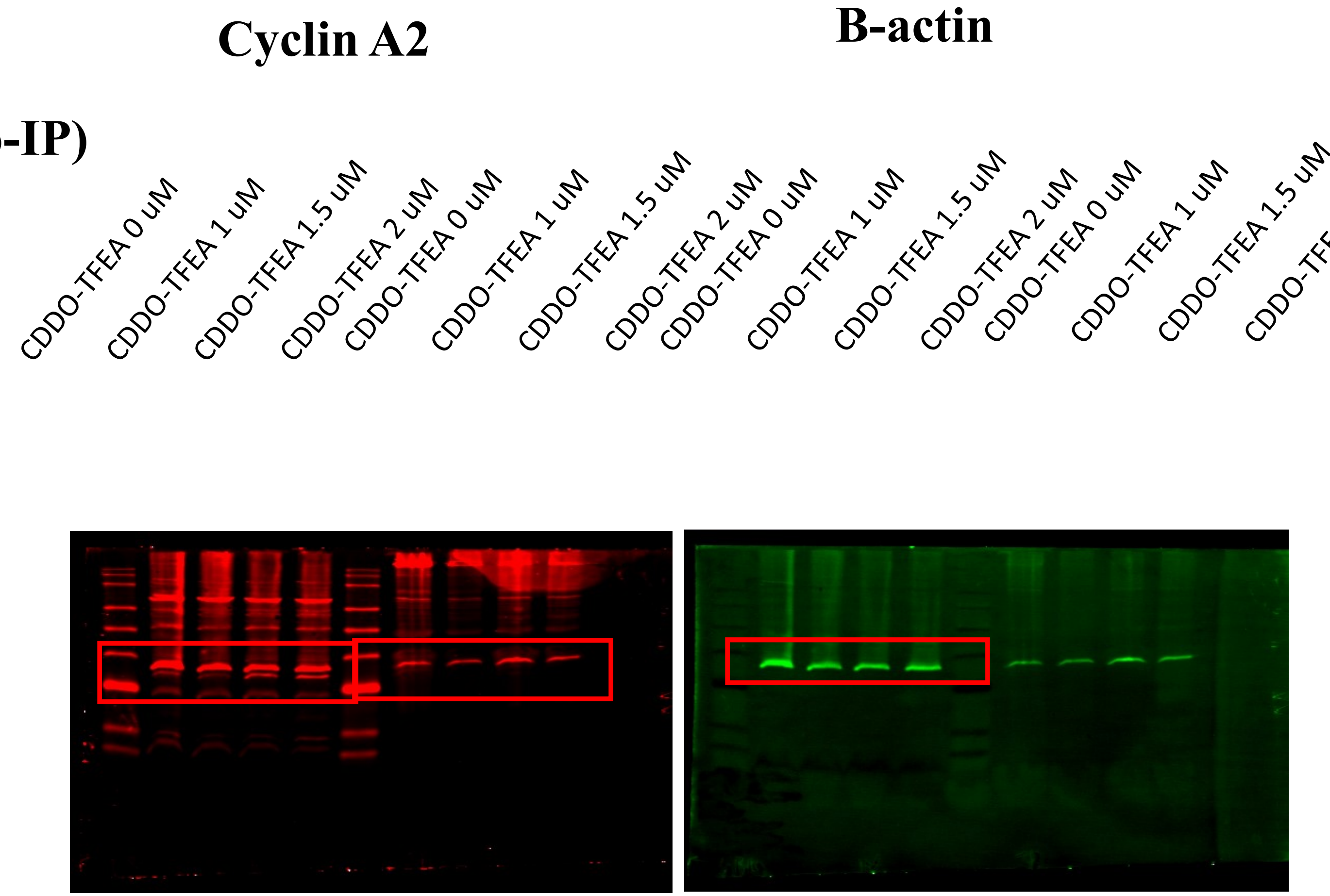

Supplement: Supplementary file 1 [file DataSheet2.PDF]
