# Supplementary material for: Induction of Mitosis Delay and Apoptosis by CDDO-TFEA in Glioblastoma Multiforme
Source: Front Pharmacol. 2021 Nov 8;12:756228. doi: 10.3389/fphar.2021.756228 (PMC8630575; doi:10.3389/fphar.2021.756228)

Supplement data

Cyclin A2/B-actin

CyclinA2

ID/Name	Ave.Intensity	Background	Backgnd SD Mult	Integ.Intensity
0-700	2508.69	0	n/a	60.54
1-700	2372.72	0	n/a	57.26
2-700	2126.51	0	n/a	51.31
3-700	2172.51	0	n/a	52.42

B-actin

ID/Name	Ave.Intensity	Background	Backgnd SD Mult	Integ.Intensity
0-800	908	0	n/a	21.91
1-800	900.19	0	n/a	21.72
2-800	877.82	0	n/a	21.18
3-800	794.37	0	n/a	19.17

CyclinA2	B-actin		
2508.69	908	2.762874	100
2372.72	900.19	2.635799	95.40061
2126.51	877.82	2.42249	87.68005
2172.51	794.37	2.734884	98.98692

Cyclin A2

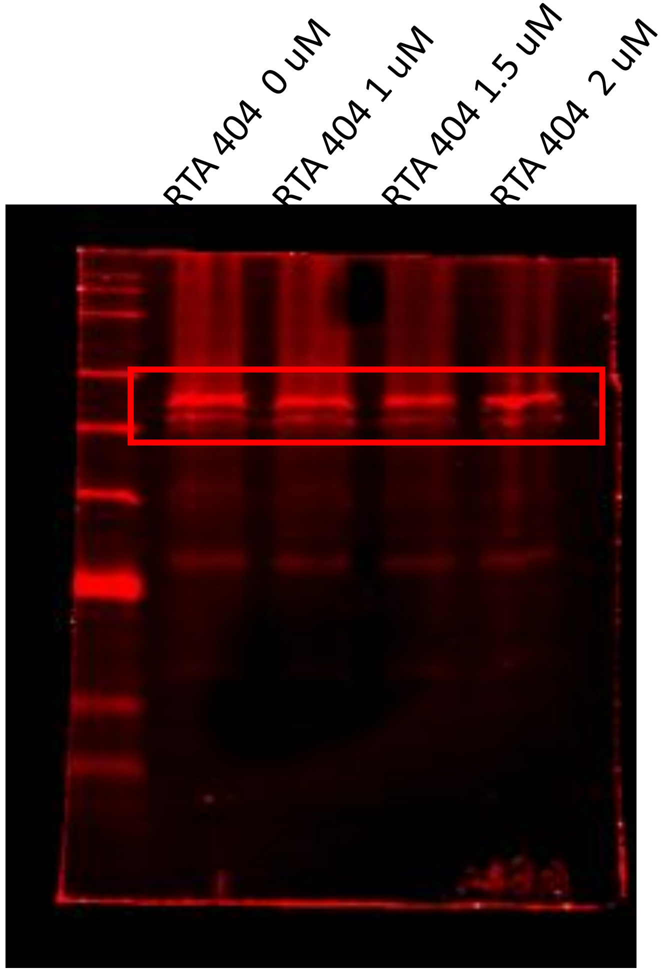

B-actin

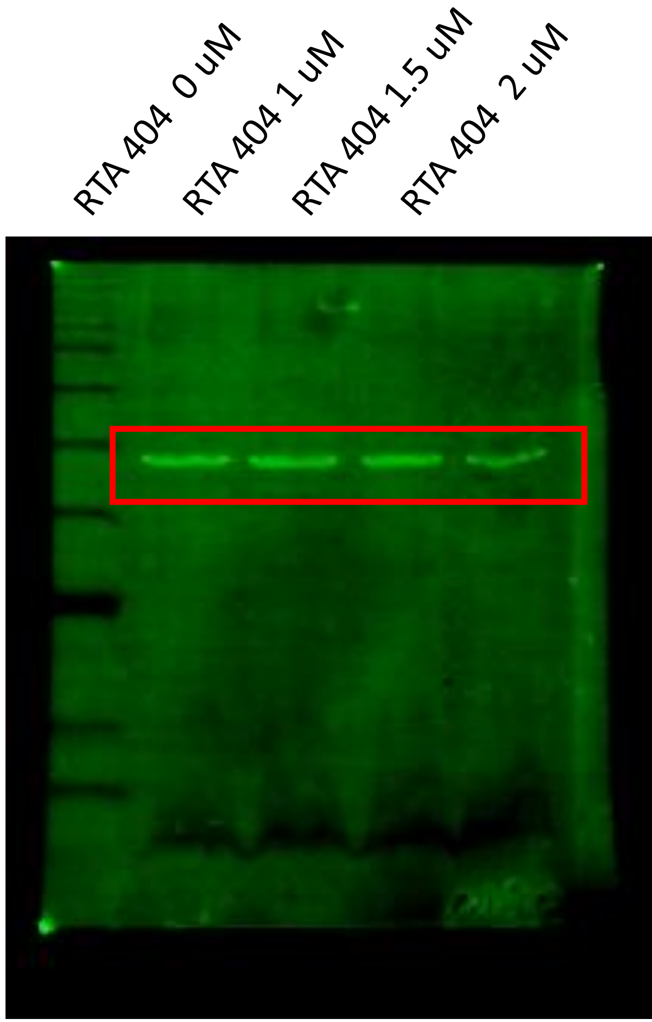

Supplement data

Cyclin B1/B-actin

CyclinB1

ID/Name	Ave.Intensity	Background	Backgnd SD Mult	Integ.Intensity
0-700	1691.02	0	n/a	23.32
1-700	1773.65	0	n/a	24.46
2-700	1902.75	0	n/a	26.24
3-700	2382.46	0	n/a	32.85

B-actin

ID/Name	Ave.Intensity	Background	Backgnd SD Mult	Integ.Intensity
0-800	738.31	0	n/a	10.18
1-800	822.46	0	n/a	11.34
2-800	895.82	0	n/a	12.35
3-800	881.6	0	n/a	12.16

CyclinB1	B-actin		%
1691.02	738.31	2.290393	100
1773.65	822.46	2.156518	94.2
1902.75	895.82	2.124032	92.7
2382.46	881.6	2.702427	118.0

Cyclin B1

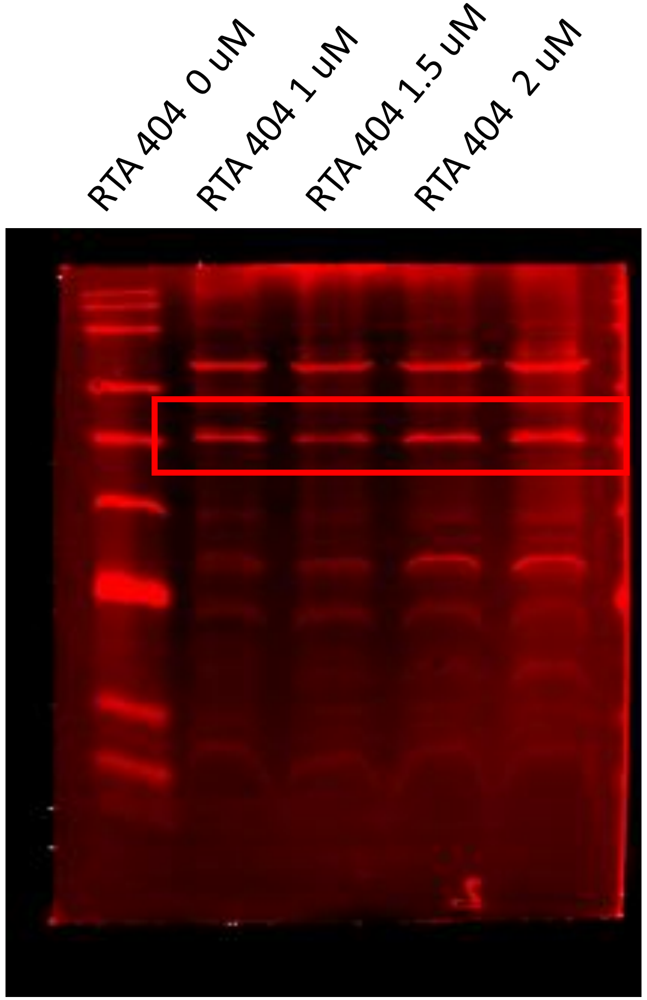

B-actin

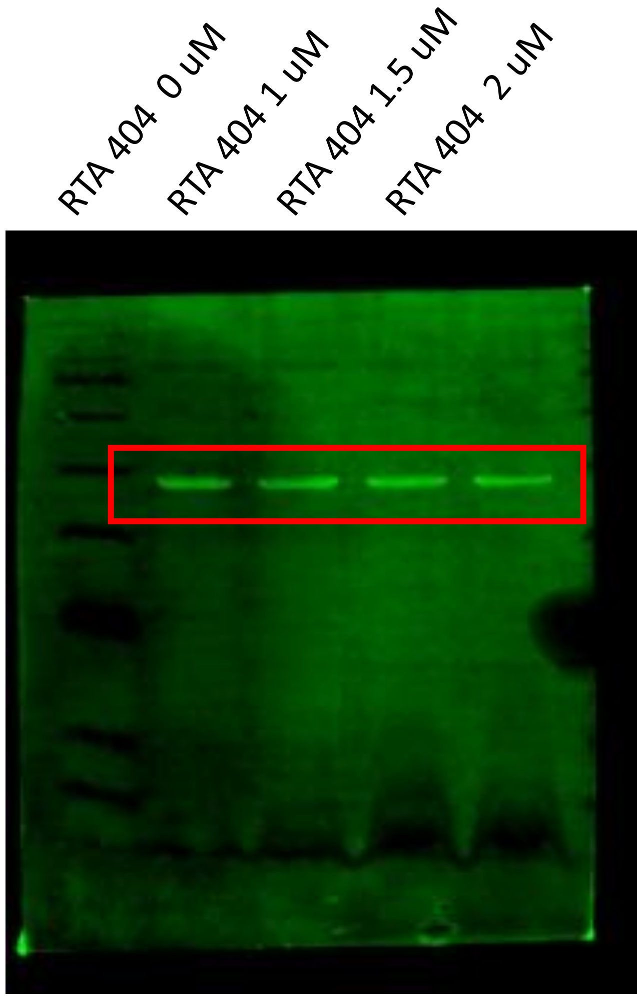

Supplement data

CDK1/B-actin

B-actin

ID/Name	Ave.Intensity	Background	Backgnd SD Mult	Integ.Intensity
0-800	483.53		0n/a	8.26
1-800	487.06		0n/a	8.33
2-800	470.99		0n/a	8.05
3-800	449.81		0n/a	7.69

CDC2

ID/Name	Ave.Intensity	Background	Backgnd SD Mult	Integ.Intensity
0-800	604.21		0n/a	10.33
1-800	478.76		0n/a	8.18
2-800	470.45		0n/a	8.04
3-800	500.51		0n/a	8.56

CDC2	B-actin	%	
604.21	483.53	1.249581	100
478.76	487.06	0.982959	78.66307
470.45	470.99	0.998853	79.93506
500.51	449.81	1.112714	89.04697

CDK1

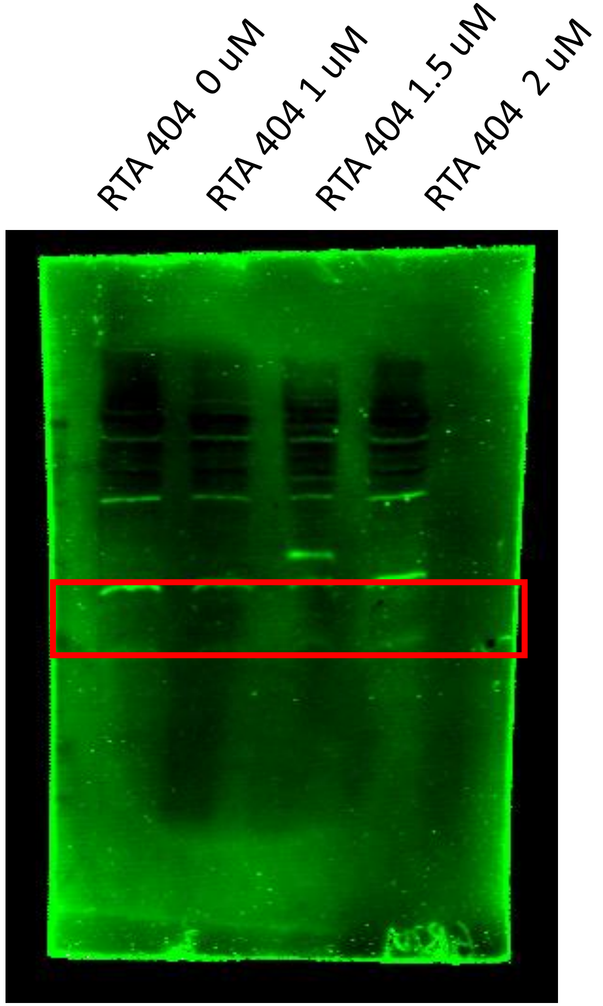

B-actin

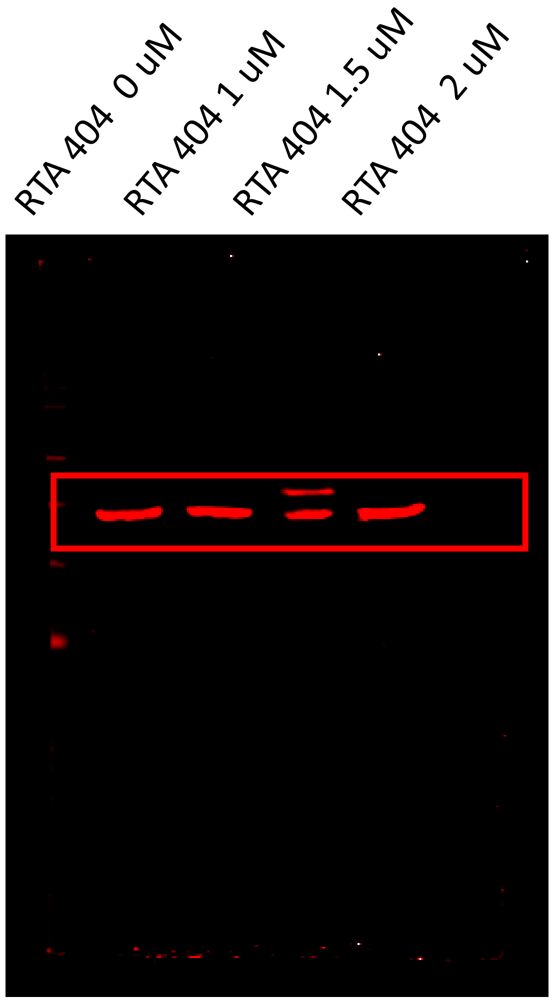

Supplement data

NRF2/B-actin

NRF2

ID/Name	Ave.Intensity	Background	Backgnd SD Mult	Integ.Intensity
0-700	1560.17	0n/a		26.89
1-700	1426.09	0n/a		24.58
2-700	1415.87	0n/a		24.4
3-700	1481.82	0n/a		25.54

B-actin

ID/Name	Ave.Intensity	Background	Backgnd SD Mult	Integ.Intensity
0-800	630.08	0n/a		10.86
1-800	676.25	0n/a		11.66
2-800	689.21	0n/a		11.88
3-800	687.03	0n/a		11.84

NRF2	B-actin		
1560.17	630.08	2.476146	100
1426.09	676.25	2.108821	85.16545
1415.87	689.21	2.054338	82.96513
1481.82	687.03	2.156849	87.10509

NRF2

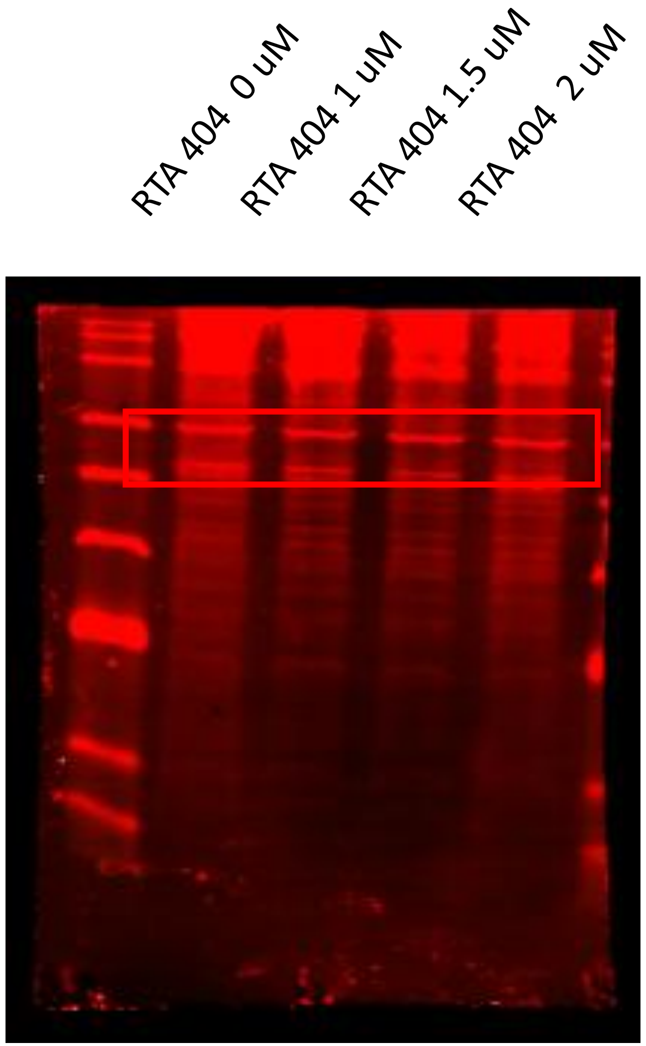

B-actin

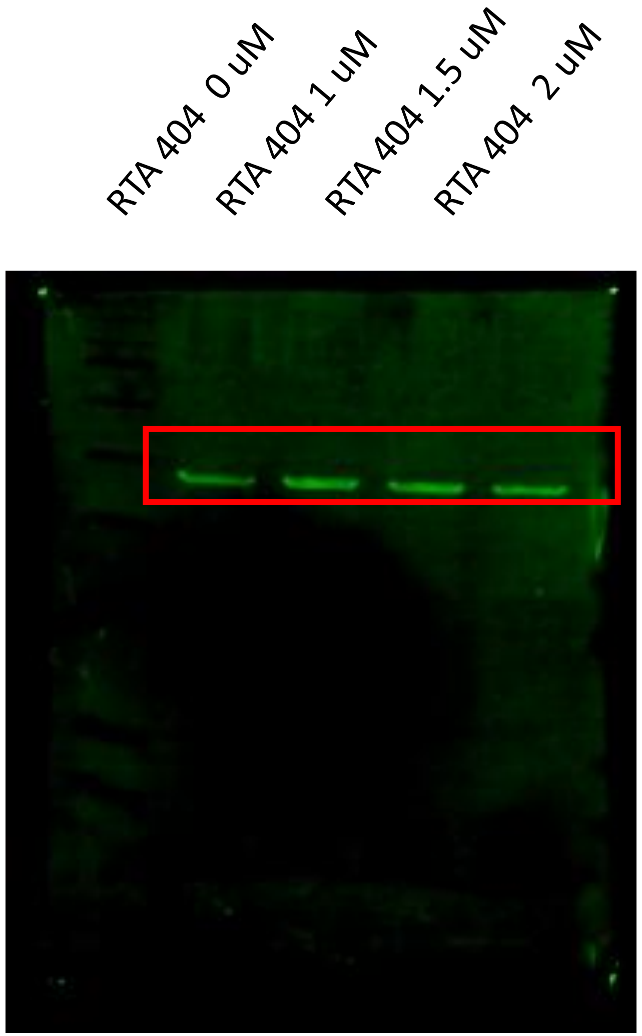

Supplement data

CHK2/p-CHK2/B-actin

Ave.Intensity	Background	Backgnd SD Mult	Integ.Intensity
417.42	0	n/a	5.01
416.44	0	n/a	5
396.1	0	n/a	4.76
349.46	0	n/a	4.2

Ave.Intensity	Background	Backgnd SD Mult	Integ.Intensity
282.37	0	n/a	3.39
268.94	0	n/a	3.23
261.94	0	n/a	3.15
259.89	0	n/a	3.12

Ave.Intensity	Background	Backgnd SD Mult	Integ.Intensity
1062.18	0	n/a	12.75
1040.59	0	n/a	12.5
1116.73	0	n/a	13.41
1144.5	0	n/a	13.74

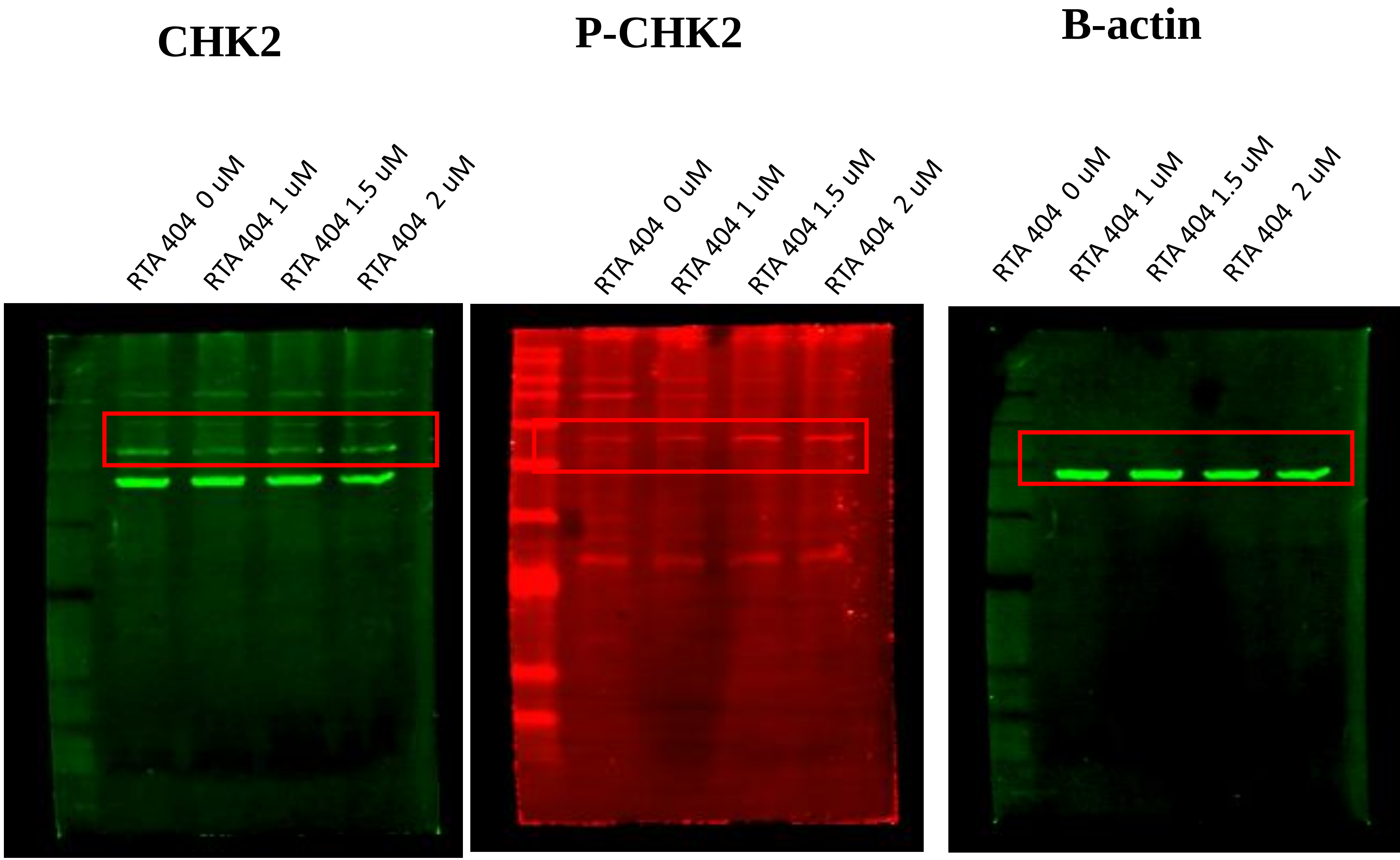

Supplement data

CHK1/B-actin

b-actin	Number	Vol. %	Volume	Height	Area
0μ M	No 1		196,007,266	65,535	5,940
1μ M	No 2		220,149,119	65,535	6,120
1.5μ M	No 3		196,885,412	65,535	6,000
2μ M	No 4		134,706,723	65,535	5,940

CHK1	Number	Vol. %	Volume	Height	Area
0μ M	No 1		82,394,099	65,535	4,895
1μ M	No 2		101,988,509	65,535	5,060
1.5μ M	No 3		100,533,628	65,535	5,060
2μ M	No 4		78,136,817	65,535	5,005

	CHk1	b-actin		%
0μ M	82,394,099	196,007,266	0.42036247	100.0
1μ M	101,988,509	220,149,119	0.46327012	110.2
1.5μ M	100,533,628	196,885,412	0.51061999	121.5
2μ M	78,136,817	134,706,723	0.58005135	138.0

CHK1

RTA 404 0 uM
RTA 404 1 uM
RTA 404 1.5 uM
RTA 404 2 uM

B-actin

RTA 404 0 uM
RTA 404 1 uM
RTA 404 1.5 uM
RTA 404 2 uM

Supplement data

p21/B-actin

b-actin	20210324A			
ID/Name	Ave.Intensity	Background	Backgnd SD Mult	Integ.Intensity
0-700	497.96	0	n/a	7.41
1-700	513.92	0	n/a	7.65
2-700	544.96	0	n/a	8.11
3-700	648.16	0	n/a	9.65
P21	20210324A			
ID/Name	Ave.Intensity	Background	Backgnd SD Mult	Integ.Intensity
0-800	321.16	0	n/a	8.76
1-800	355.93	0	n/a	9.71
2-800	379.52	0	n/a	10.36
3-800	453.3	0	n/a	12.37

	P21	b-actin		%
0μ M	321.16	497.96	0.6449514	100
1μ M	355.93	513.92	0.69257861	107.4
1.5μ M	379.52	544.96	0.69641809	108.0
2μ M	453.3	648.16	0.69936435	108.4

p21

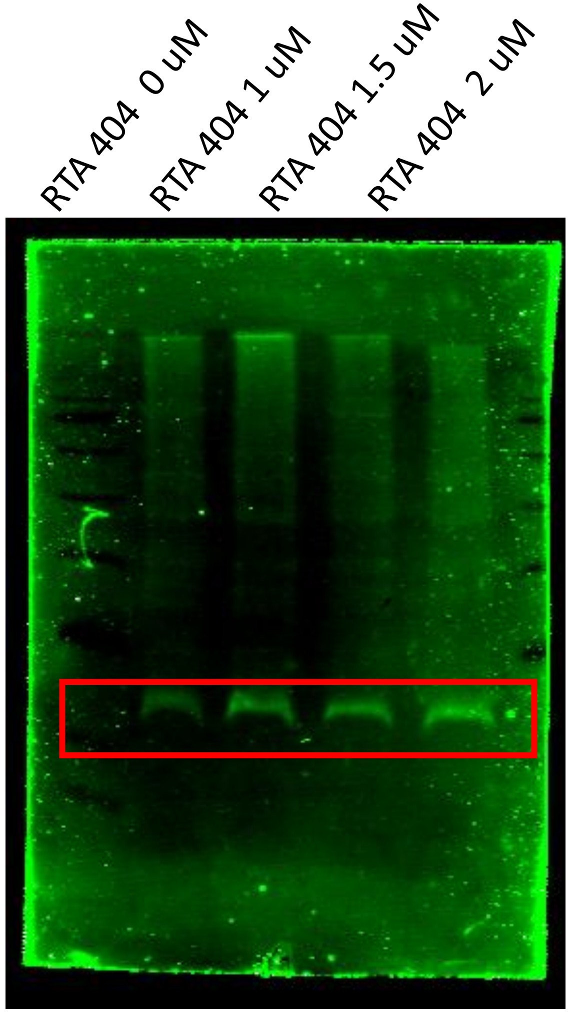

B-actin

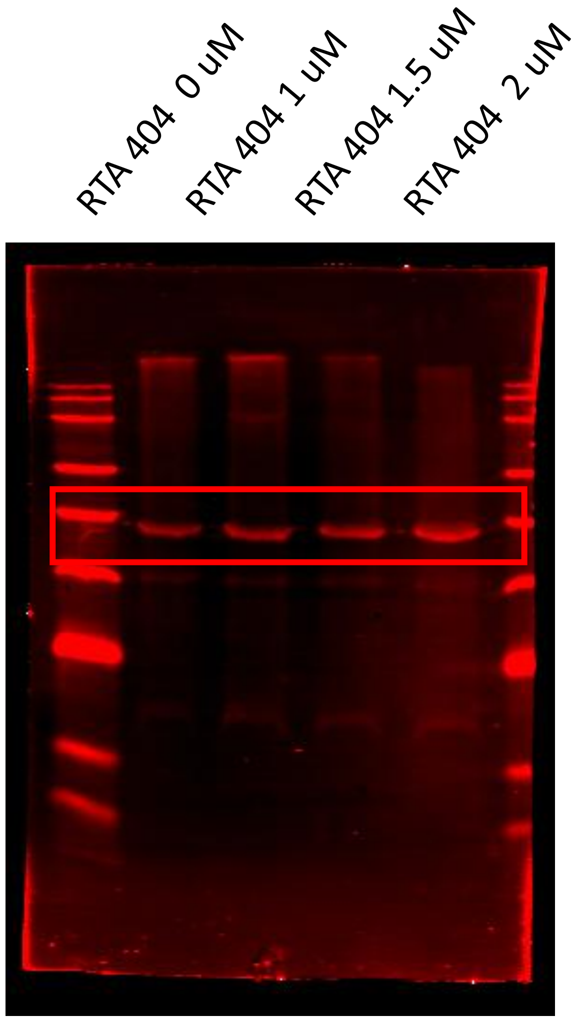

Supplement data

Raw data

Figure 8 Co-immunoprecipitation (Co-IP)

CyclinB1

ID/Name	Ave.Intensity	Background	Backgnd SD Mult	Integ.Intensity
0-700	2381.7	0n/a		42.69
1-700	2250.62	0n/a		40.34
2-700	2119.57	0n/a		38
3-700	2159.65	0n/a		38.71
4-700	1683.6	0n/a		30.18
5-700	1276.2	0n/a		22.88
6-700	1589.36	0n/a		28.49
7-700	1576.74	0n/a		28.26

CDK1

ID/Name	Ave.Intensity	Background	Backgnd SD Mult	Integ.Intensity
0-800	566.79	0n/a		11.07
1-800	471.71	0n/a		9.21
2-800	453.73	0n/a		8.86
3-800	482.39	0n/a		9.42
4-800	363.3	0n/a		7.1
5-800	346.38	0n/a		6.77
6-800	403.5	0n/a		7.88
7-800	388.22	0n/a		7.58

	CyclinB1	CDK1		CyclinB1/CDK1
0μ M	1683.6	363.3	4.634187	100
1μ M	1276.2	346.38	3.684393	79.50463
1.5μ M	1589.36	403.5	3.938934	84.99732
2μ M	1576.74	388.22	4.06146	87.64127

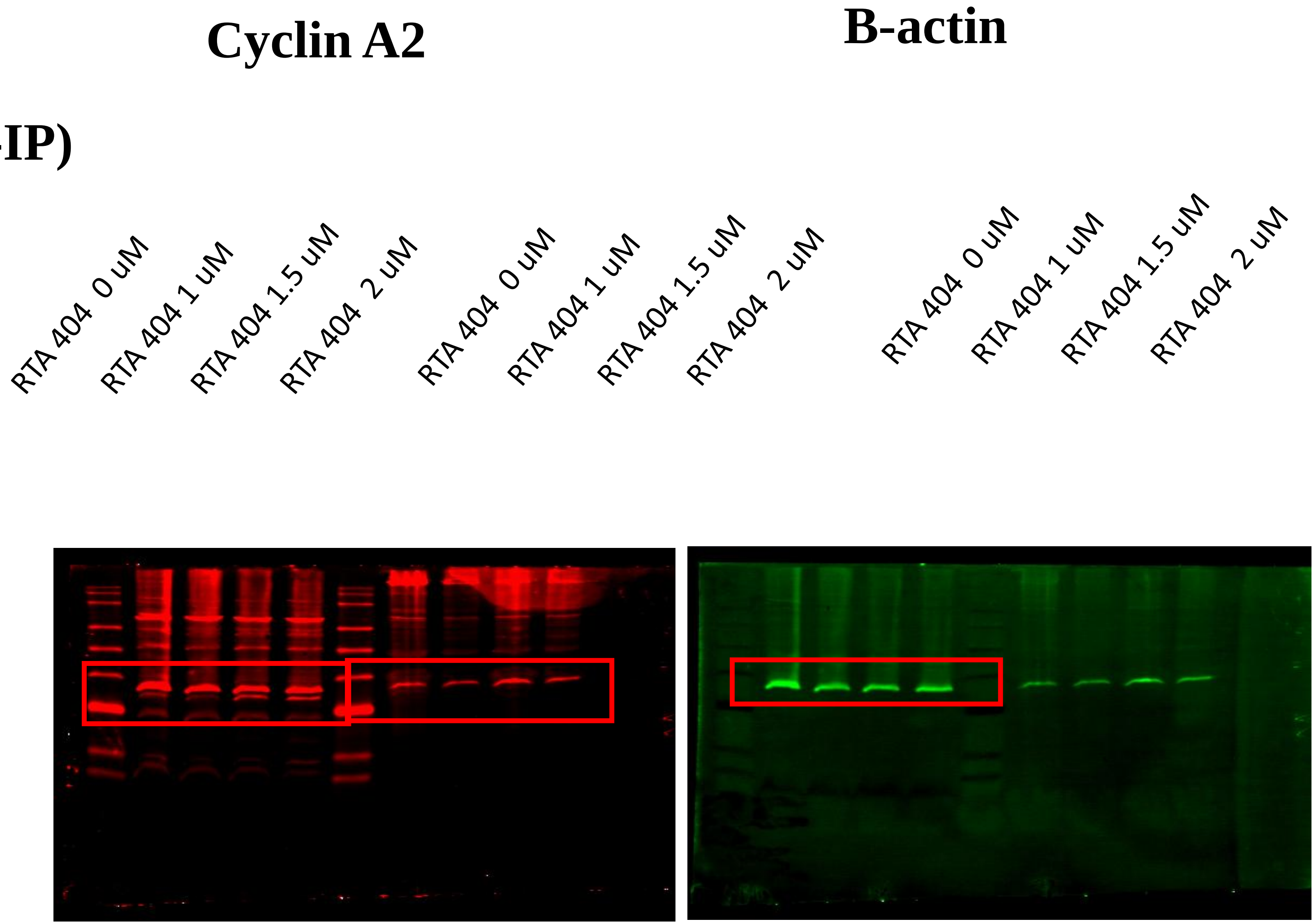

Supplement: Supplementary file 3 [file DataSheet3.PDF]
